# An open‐source deep learning framework for respiratory motion monitoring and volumetric imaging during radiation therapy

**DOI:** 10.1002/mp.18015

**Published:** 2025-07-15

**Authors:** Nicholas Hindley, Paul J. Keall

**Affiliations:** ^1^ Image X Institute School of Health Sciences Faculty of Medicine and Health University of Sydney Sydney NSW Australia

**Keywords:** deep learning, image‐guided radiation therapy, motion management, MRI, real‐time tracking, x‐ray

## Abstract

**Background:**

Real‐time image‐guided radiation therapy (IGRT) was first clinically implemented more than 25 years ago but is yet to find widespread adoption. Existing approaches to real‐time IGRT require dedicated or specialized equipment that is not available in most treatment centers and most techniques focus exclusively on targets without tracking the surrounding organs‐at‐risk (OARs).

**Purpose:**

To address the need for inexpensive real‐time IGRT, we developed *Voxelmap*, a deep learning framework that achieves 3D respiratory motion estimation and volumetric imaging using the data and resources already available in standard clinical settings. This framework can also be adapted to other imaging modalities such as MRI‐Linacs. In contrast with existing approaches, which constrain the solution space with linear priors, *Voxelmap* encourages diffeomorphic mappings that are topology‐preserving and invertible.

**Methods:**

Deformable image registration and forward‐projection or slice extraction were used to generate patient‐specific training datasets of 3D deformation vector fields (DVFs) and 2D images (or k‐space data) from pretreatment 4D‐CT or 4D‐MRI scans. The XCAT and CoMBAT digital phantoms and SPARE Grand Challenge Dataset provided synthetic and patient data, respectively. Five network architectures were used to predict 3D DVFs from 2D imaging data. Networks A‐C were trained on x‐ray images, Network D was trained on MR images and Network E was trained on k‐space data. Using *Voxelmap*, network‐generated 3D DVFs were used to warp both structures contoured on the peak‐exhale pretreatment image and the image itself to enable simultaneous target and OAR tracking and volumetric imaging. Using the standard‐of‐care approach, contours were expanded to internal target volumes.

**Results:**

Validating on digital phantom data for x‐ray guided treatments of cardiac arrhythmia, mean Dice similarity between predicted and ground‐truth target and OAR contours for Networks A‐C ranged from 0.81 ± 0.05 to 0.82 ± 0.05 and 0.78 ± 0.04 to 0.81 ± 0.04, respectively, while target centroid error ranged from 2.0 ± 0.5 to 2.3 ± 0.9 mm. For MRI‐based digital phantom data, mean Dice similarity for target and OAR contours was 0.91 ± 0.06 and 0.90 ± 0.02 for both Networks D and E, while target centroid error ranged from 1.7 ± 0.8 to 1.8 ± 0.8 mm. For x‐ray‐based lung cancer patient data, mean Dice similarity for target and OAR contours for Networks A‐C ranged from 0.86 ± 0.05 to 0.89 ± 0.04 and 0.94 ± 0.01 to 0.97 ± 0.01, respectively. However, in terms of target centroid error, only Network A outperformed an ITV‐based approach at 1.8 ± 0.7 mm while Networks B and C exhibited large errors of 2.7 ± 1.2 to 3.5 ± 1.4 mm, respectively. Target volumes dynamically shifted using *Voxelmap* were 31 % smaller than the standard‐of‐care.

**Conclusions:**

*Voxelmap* provides a generalized, open‐source tool for intrafraction respiratory motion monitoring and volumetric imaging. Comparing tracking errors across synthetic and patient data revealed that certain network architectures are more robust to the scatter and noise profiles encountered in typical clinical settings. These learnings will inform future developments in real‐time motion tracking. Our code is available at https://github.com/Image‐X‐Institute/Voxelmap .

## 1 INTRODUCTION

Cancer is one of the leading causes of death worldwide and radiation therapy remains one of the most clinically beneficial and cost‐effective treatments.^1^ In fact, it is estimated that one in every two cancer patients can benefit from radiation therapy.^2^ Additionally, there is a growing interest in non‐nvasively treating cardiac arrhythmias with radiation therapy.^3^, ^4^, ^5^ At the level of treatment delivery, the ultimate goal of radiation therapy is to deliver a therapeutic dose to the target while minimizing damage to the surrounding organs‐at‐risk (OARs). However, intrafraction motion (i.e. motion encountered during treatment) makes these competing goals difficult to achieve. Indeed, respiratory‐induced motion of several centimeters has been reported for lung,^6^, ^7^, ^8^, ^9^ liver,^10^, ^11^, ^12^, ^13^, ^14^ and pancreatic^15^, ^16^, ^17^ tumors. To overcome the challenge of intrafraction motion, real‐time image‐guided radiation therapy (IGRT) was proposed more than 25 years ago by Shirato et al.^18^ and many real‐time IGRT techniques have been developed since then, including the CyberKnife,^19^ Vero,^20^ Calypso,^21^ and RayPilot^22^ systems as well as ultrasound^23^ and MR guidance.^24^, ^25^ However, the vast majority of these techniques focus exclusively on targets without monitoring the surrounding OARs. Moreover, all of these approaches require dedicated or specialized equipment that is not available in most treatment facilities. A recent survey of 200 radiation therapy centers across 41 countries found that 71% wished to offer real‐time respiratory motion management to more patients but were limited by resources.^26^ Software solutions to real‐time IGRT could address this global demand by offering inexpensive ways to track targets and OARs.

### 1.1 Existing approaches

Given the available hardware on contemporary radiation therapy systems, 3D‐IGRT involves the ill‐posed problem of mapping from 2D images to 3D motion. Broadly speaking, this problem can be addressed in three ways. First, motion models can be used to capture geometric correspondences between surrogate signals (including images) and 3D motion. Second, image reconstruction with sparse sampling can be used to generate 3D images from 2D images and the desired 3D deformation vector field (DVF) can then be produced via 3D‐3D image registration to a reference 3D image. Third, 2D‐3D image registration can be performed directly between an acquired 2D image and a 3D reference image to produce the desired 3D DVF. Once this 3D DVF is known it can then also be used to warp the reference image to enable volumetric imaging. It should be noted that any proposed solution must be implementable in real‐time. As a guideline, the AAPM Task Group 264 suggest a threshold of 500 milliseconds for the safe clinical implementation of MLC tracking.^27^ Therefore, taking into account image acquisition (e.g. ∼100 milliseconds for x‐ray imaging at 10 Hz) and motion compensation (e.g. ∼110 milliseconds for MLC tracking), computation times of 300 milliseconds or less become crucial.

#### 1.1.1 Motion models

A motion model can be defined as a process that takes some surrogate data as input and produces a motion estimate as output.^28^ Among the sources of intrafraction motion, respiratory‐induced motion has been the most studied owing to the large displacements of a centimeter or more and its broad influence across the thorax and abdomen.^29^ Seminal work by Zhang et al. found that two principal components accounted for up to 90% of the variance of 3D DVFs computed from 4D‐CT scans^30^. Since then a number of principal component analysis (PCA)‐based techniques have been used to estimate 3D motion from both x‐ray^31^, ^32^, ^33^‐ and MR^34^, ^35^, ^36^‐derived signals. The compressibility of 3D respiratory motion fields to a few principal components reflects the strong spatial correlations of these fields. However, it is difficult to adapt these models to the *interfraction* changes that occur between planning and treatment. We found that even using pretreatment imaging to update motion models, a PCA‐based approach still produced unrealistic organ deformation and large localization errors.^37^ Moreover, an inherent limitation of PCA is that it only captures linear relationships between variables, which does not reflect the underlying nonlinear reality of anatomical variation.

#### 1.1.2 Sparse sampling image reconstruction

Common to the problems of image reconstruction and image registration is the need to confine the solution space via regularization due to a lack of unique solutions. Additionally, the theoretically achievable limits of sparse sampling are determined by the Shannon–Nyquist theorem,^34^ beyond which image artefacts become unavoidable. To address these issues, previously reported compressed sensing methods have leveraged total variation, spatial‐temporal correlations,^39^ known subregions,^40^ and L1 regularization.^41^, ^42^ However, where ultrasparse sampling is used, these techniques fail to produce the high‐quality images required for interventions such as image‐guided radiation therapy. Pioneering work by Shen et al. pushed sparse sampling to the limit by using a deep neural network to perform patient‐specific 3D image reconstruction from a single 2D image.^43^ However, even with input 2D images that were downsampled to 128 × 128, this approach required a large network with approximately half a billion trainable parameters. Additionally, a new network needs to a be trained on a new dataset for each unique angle,^44^ demanding even greater training times and memory requirements for realistic treatment scenarios. Furthermore, inference times for the image reconstruction task alone exceeded the AAPM threshold of 500 milliseconds.

#### 1.1.3 2D‐3D image registration

2D‐3D image registration denotes the problem of finding an appropriate transformation between a 2D image and a 3D image given a shared coordinate space. Existing approaches can be classed as calibration‐based, extrinsic, or intrinsic.^45^ However, calibration‐based methods require precalibrated equipment and integrated 2D/3D systems that are not available in most clinical settings.^46^, ^47^, ^48^ Extrinsic methods use markers implanted in bone,^49^, ^50^ soft tissue,^51^, ^52^, ^53^, ^54^ or skin^55^, ^56^ with known positions to track 3D motion during 2D imaging but this involves invasive surgery, increases the risk of complications^57^ and may suffer from drift^58^ or surrogacy errors.^59^, ^60^, ^61^, ^62^, ^63^ Finally, intrinsic methods perform the registration task directly between acquired and reference images but most existing intrinsic registration algorithms compute rigid motion^64^, ^65^, ^66^, ^67^, ^68^, ^69^, ^70^, ^71^, ^72^, ^73^, ^74^, ^75^ while relatively few account for nonrigid organ deformation. Image registration is also often achieved iteratively making most methods unsuitable for real‐time implementation. Moreover, existing machine learning approaches to nonrigid 2D‐3D image registration use PCA to impose priors on the solution space.^76^, ^77^, ^78^

### 1.2 Deep learning‐based 3D respiratory motion modelling and volumetric imaging

Following the pioneering work of Shen mentioned earlier, recent work has explored the reconstruction of 3D anatomical information from sparse 2D imaging data via deep neural networks. Loyen et al. adapted a CNN architecture for online 3D reconstruction from single digitally reconstructed radiographs (DRRs),^79^ while Zhang et al. employed a two‐stage CNN for digital‐radiograph‐only carbon‐ion therapy planning.^80^ To improve reconstruction from multiple projections, Chang et al. proposed InverseNet3D, using separate feature extractors for two orthogonal x‐ray images,^81^ and Lei et al. developed a feature matching network incorporating geometric realignment.^82^ More recently, Shao et al. and Dai et al. addressed the challenge of arbitrary‐angle projection reconstruction for joint reconstruction and motion estimation.^83^, ^84^ Although these approaches demonstrate the potential to reconstruct 3D volumes from limited data, they often rely on specialized projection geometries, require long inference times or constrain the solution space through linear priors or rigid model assumptions.

**FIGURE 1:**
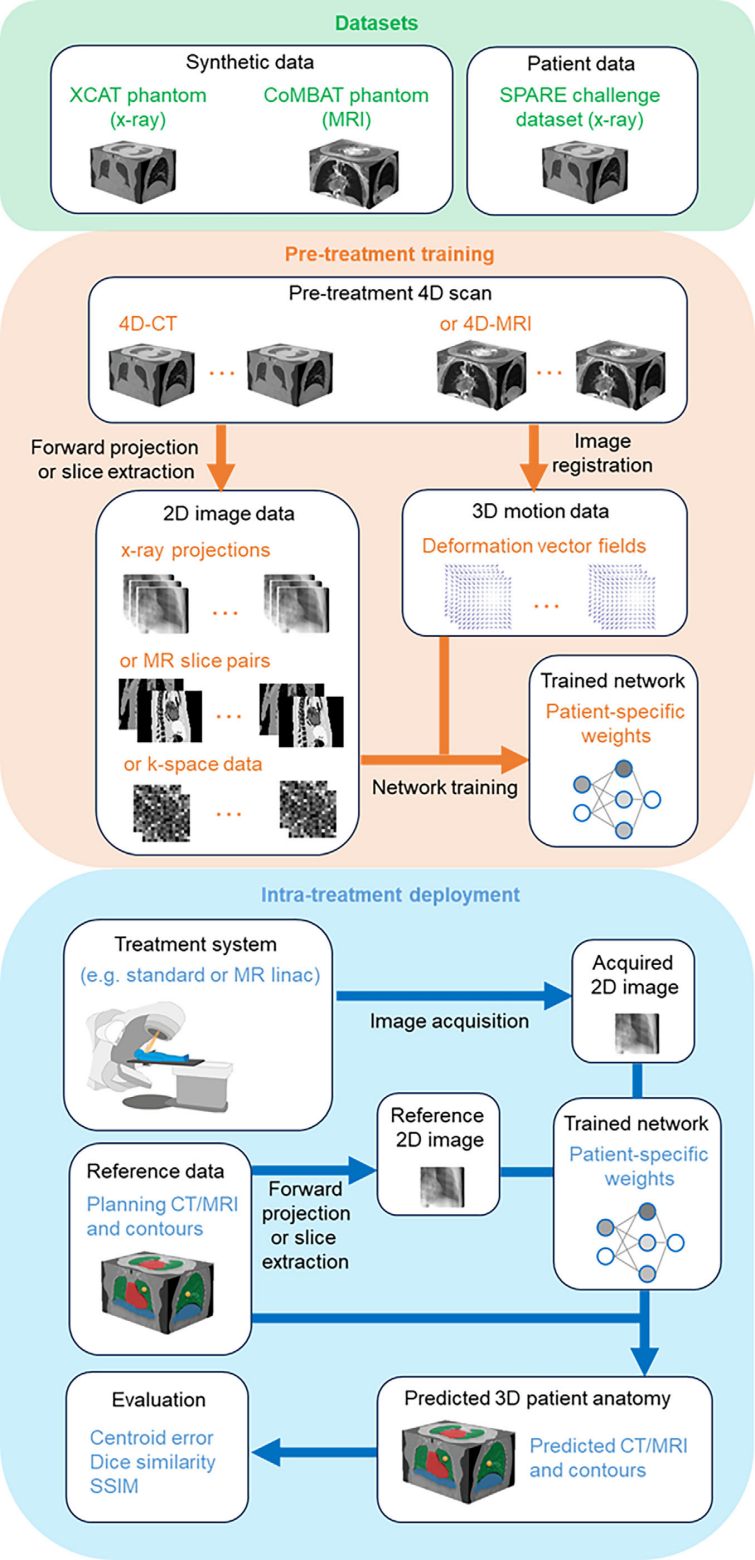
Overview of method developed and evaluated in this paper. XCAT means extended cardiac‐torso; CoMBAT means CT/MRI breathing XCAT; SPARE means sparse‐view reconstruction; CT means computed tomography; MRI means magnetic resonance image; linac means linear accelerator; and SSIM means structural similarity index measure.

Here we advance a deep learning framework for 3D respiratory motion modelling and volumetric imaging called *Voxelmap* (Figure 1). In this framework, a mapping between intrafraction images and DVFs was learned directly using data acquired in standard clinical workflows and can be deployed using the available equipment in the bulk of radiation therapy centers around the world. Additionally, as new techniques are developed, *Voxelmap* can be adapted to different image modalities. Using pre‐treatment 4D imaging data (e.g. 4D‐CT), conventional 3D‐3D image registration was performed between a reference 3D image and every other 3D image to produce the desired 3D DVFs. Additionally, forward projection or slice extraction from the 3D images was used to produce the desired 2D images (or k‐space data) for training. Once the neural network is trained, 2D images acquired during treatment were registered to a reference 2D or 3D image to produce the desired 3D DVF. Additionally, this DVF was used to transform a reference 3D image to enable real‐time volumetric imaging. It should be noted that here we introduce a framework that is agnostic to network architecture. The key idea is that patient‐specific image‐motion mappings can be learned using the available data in image‐guided radiotherapy with deep neural networks. Here, inspired by Shen et al.,^43^ a residual network was used to produce a latent, low‐dimensional feature map of the input images through an encoding arm that is then decoded to generate the desired 3D DVF.

In contrast with existing approaches that confine the solution space with PCA‐based priors, here we consider solutions in the space of diffeomorphisms and single flow velocity fields that remain constant over time.^85^ More specifically, for diffeomorphic mapping φ and stationary velocity field u, we consider the ordinary differential equation

(1) $$\frac{\partial \varphi^{\left(t\right)}}{\partial t}=u\left(\varphi^{\left(t\right)}\right)$$

where $\varphi^{\left(0\right)}$ is the identity mapping (i.e. that where everything remains stationary) and integration over the interval t=[0,1] yields $\varphi^{\left(1\right)}$ (i.e. the DVF which maps from t=0 to t=1). Inspired by Dalca et al.^86^, integration is achieved numerically by *squaring and scaling* ^87^ in the final layer of the network using. Our choice to encourage diffeomorphic transformations is based on their desirable properties as topology‐preserving and invertible mappings.

### 1.3 Contribution

We initially established proof‐of‐concept for estimating 3D motion from 2D images in a technical note^88^ using x‐ray data from two lung cancer patients. In the present work, we introduce a generalized framework and extend beyond these initial results substantially by:

1. Demonstrating that *Voxelmap* can be flexibly deployed across image modalities (x‐ray, MRI) and treatment sites (lung, heart).
2. Introducing and comparing a range of new neural network architectures.
3. Validating *Voxelmap* using the XCAT and CoMBAT digital phantoms for which there are known positions for every anatomical structure.
4. Demonstrating that *Voxelmap* produces invertible, topology‐preserving mappings.
5. Performing new in silico experiments to better understand learned representations.
6. Providing *Voxelmap* as an open‐source repository.

By testing our proposed framework in this manner, we offer a general framework for real‐time respiratory motion monitoring and volumetric imaging during image‐guided radiation therapy.

## 2 METHODS AND MATERIALS

### 2.1 Problem formulation

**FIGURE 2:**
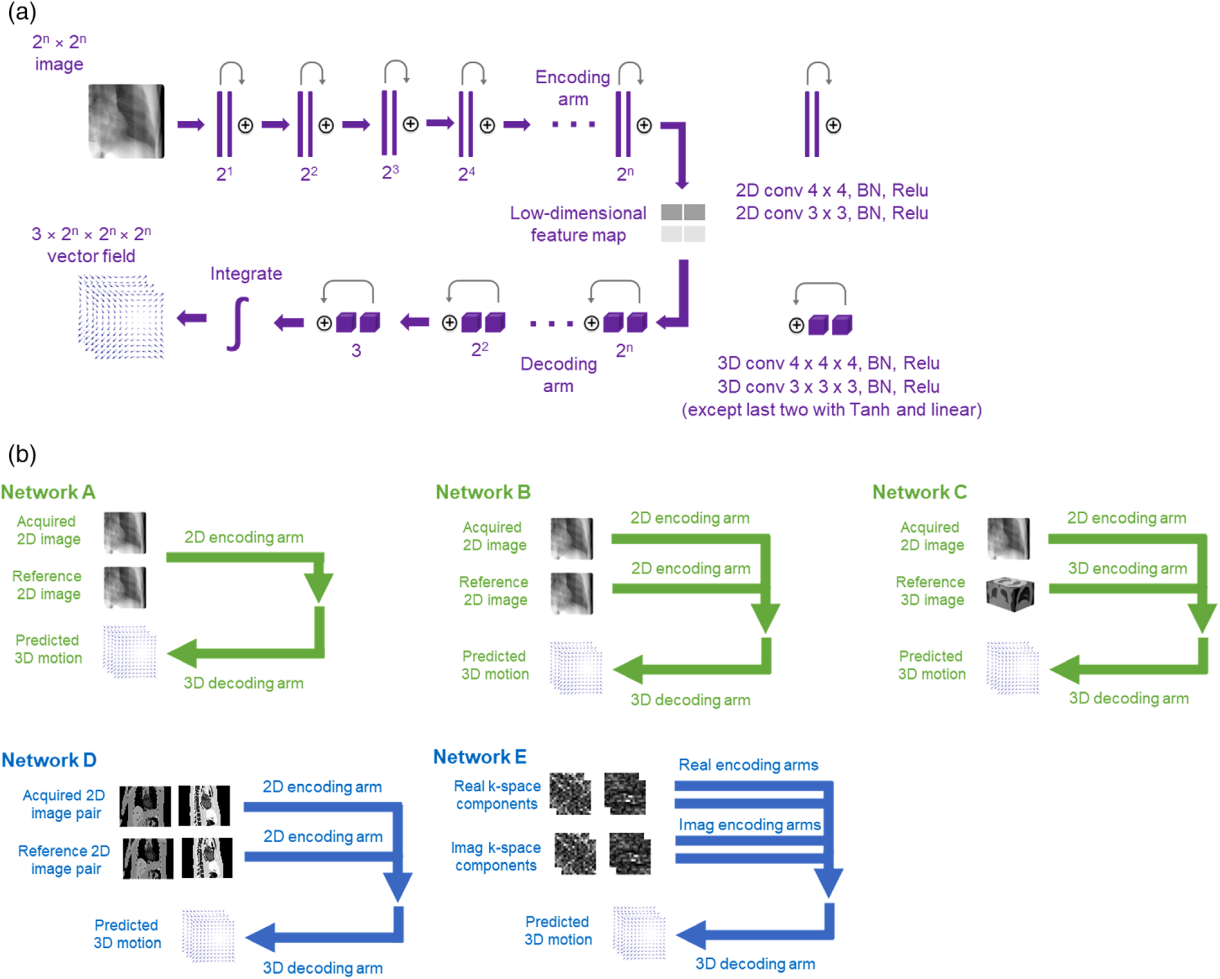
(a) General arrangement of residual layers for networks validated in this paper with the number of output channels shown below layers. (b) Different architectures leveraging these layers to predict 3D motion from x‐ray and magnetic resonance imaging data.

Here we formulate the task of mapping between intrafraction images and 3D DVFs in a variety of ways. However, in every formulation, a neural network N with parameters θ is used to encode features from some acquired and reference images (or k‐space data), which are then decoded to predict a 3D DVF (Figure 2a). Additionally, we suppose that this DVF can be represented by diffeomorphic mapping φ such that X·φ=Y, where X and Y are reference and acquired 3D images, respectively. Given these 3D images, there are a number of related 2D images. In the case of x‐ray imaging, we consider $x_{\alpha }$ and $y_{\alpha }$ which are acquired by forward‐projecting X and Y, respectively, at angle α. Similarly, in the case of magnetic resonance imaging (MRI), we consider 2D image pairs $\left\{x_{c},x_{s}\right\}$ and $\left\{y_{c},y_{s}\right\}$ which are coronal and sagittal slices of X and Y, respectively. Deciding how and which 2D and 3D images are used to predict φ yields a range of different neural network architectures and training regimes. Networks A, B, and C below were used for x‐ray experiments, while Networks D and E were used for MRI experiments (Figure 2b).

#### 2.1.1 Network A

Acquired and reference 2D images are concatenated as input to an encoding arm to generate a feature map which is then decoded to yield the predicted DVF $\hat{\varphi }$ as output, such that $N\;\left(x_{\alpha },y_{\alpha };\;\theta \right)=\hat{\varphi }$. This network is motivated by the observations that 2D‐2D image registration can be achieved with deep neural networks by using concatenated 2D images as input^89^, ^90^ and that 2D views provide hints about 3D motion. For instance, inhalation involves simultaneous inferior and anterior motion of the diaphragm such that an inferior shift in the coronal plane corresponds to an anterior shift in the sagittal plane. In other words, certain “allowed” patterns of 3D motion emerge given the corresponding 2D views. As instantiated in this work, Network A had 10 836 973 trainable parameters. Inference time for Network A was 19 milliseconds.

#### 2.1.2 Network B

Acquired and reference 2D images are encoded separately to generate independent feature maps that are concatenated and decoded to yield the predicted DVF $\hat{\varphi }$ as output, such that $N\;\left(x_{\alpha },y_{\alpha };\;\theta \right)=\hat{\varphi }$. This network is motivated by the idea that learned representations may benefit from encoding acquired and reference images differently. As instantiated in this work, Network B had 4 130 925 trainable parameters. Inference time for Network B was 21 milliseconds.

#### 2.1.3 Network C

Acquired 2D image $x_{\alpha }$ and reference 3D image Y are encoded separately to generate independent feature maps that are concatenated and decoded to yield the predicted DVF $\hat{\varphi }$ as output, such that $N\;\left(x_{\alpha },Y;\;\theta \right)=\hat{\varphi }$. This network is motivated by the idea that there may be additional information in Y that is lost in using the forward‐projection $y_{\alpha }$ as input. As instantiated in this work, Network C had 5 048 373 trainable parameters. Inference time for Network C was 21 milliseconds.

#### 2.1.4 Network D

Acquired and reference 2D image pairs $\left\{x_{c},x_{s}\right\}$ and $\left\{y_{c},y_{s}\right\}$ are each concatenated and encoded separately to generate independent feature maps that are then concatenated and decoded to yield the predicted DVF $\hat{\varphi }$ as output, such that $N\;\left(x_{c},x_{s},y_{c},y_{s};\;\theta \right)=\hat{\varphi }$. This network is an analog to Network B for MR images. As instantiated in this work, Network D had 16 516 901 trainable parameters. Inference time for Network D was 21 milliseconds.

#### 2.1.5 Network E

The real components of k‐space data corresponding to acquired and reference coronal $\left\{x_{c}^{real},y_{c}^{real}\right\}$ and sagittal $\left\{x_{s}^{real},y_{s}^{real}\right\}$ slices are each concatenated and encoded to generate independent feature maps that are then added. Similarly, the imaginary components of k‐space data corresponding to acquired and reference coronal $\left\{x_{c}^{imag},y_{c}^{imag}\right\}$ and sagittal $\left\{x_{s}^{imag},y_{s}^{imag}\right\}$ slices are also concatenated, encoded, and added. These real and imaginary feature maps are then concatenated and decoded to yield the predicted DVF $\hat{\varphi }$ as output, such that $N\;\left(x_{c}^{real},y_{c}^{real},x_{s}^{real},y_{s}^{real},x_{c}^{imag},y_{c}^{imag},x_{s}^{imag},y_{s}^{imag}\;;\;\theta \right)=\hat{\varphi }$. This network extends Network D to handle k‐space data (without direct access to images). As instantiated in this work, Network E had 19 489 717 trainable parameters. Inference time for Network E was 23 milliseconds.

### 2.2 Data

#### 2.2.1 XCAT phantom (*n* = 8)

Imaging data for x‐ray‐guided cardiac radioablation (i.e. noninvasive treatment of cardiac arrhythmia with radiation therapy) were generated using the 4D extended cardiac‐torso (XCAT) phantom,^91^ as described previously.^92^ Briefly, traces from the combined measurement of ECG, Breathing and Seismocardiogram (CEBS) database^93^ were used to program cardiorespiratory motion for eight phantoms with unique anatomies. Since the respiratory data were acquired as relative signals using a piezoresistive band, each phantom was assigned with maximum diaphragm motion amplitude set to either 5 (for Phantoms 1–4) or 10 (for Phantoms 5–8) mm to produce target motion within clinically observed ranges.^94^, ^95^ Similarly, ECG traces were used to deform a 3D nonuniform rational B‐spline surface of the heart to simultaneously produce cardiac motion.

To generate training data, 4D‐CT scans were simulated by segmenting each trace into 10 respiratory bins and generating a 3D image for each bin. Each 3D image was then forward‐projected at 630 imaging angles over one 360⁰ arc to yield a total of 6300 2D images as digitally‐reconstructed radiographs (DRRs). To generate testing data, a 1 min section from each trace was used to simulate intrafraction imaging by generating volumes at a rate of 10.5 Hz and forward‐projecting each volume once to produce 630 DRRs over one 360⁰ arc.

All volumes were originally generated with voxel sizes of 1 mm × 1 mm × 1 mm and dimensions 512 × 256 × 512. However, due to memory constraints, they were then downsampled to dimensions 128 × 128 × 128, yielding voxel sizes of 4 mm × 2 mm × 4 mm. Similarly, all projections were generated with pixel sizes of 0.388 mm × 0.388 mm and dimensions 1024 × 768 but were downsampled to dimensions 128 × 128 to yield pixel sizes of 3.1 mm × 2.3 mm. It should be noted that this downsampling was performed due to hardware constraints and does not reflect a fundamental limitation of the method. Therefore, the results reported here represent a lower limit in terms of performance as *Voxelmap* can be scaled to accommodate higher resolution images as computational technology advances.

As the position of every structure in the thorax and abdomen is known precisely, contours for the target and organs‐at‐risk were defined by selecting the voxels corresponding to the underlying structures. Following our previous work on cardiac ablation, here we selected the left atrium as the target.^92^ Following the ENCORE‐VT trial,^96^ we selected the stomach, esophagus, lungs, and spinal cord as the organs‐at‐risk.

#### 2.2.2 CoMBAT phantom (*n* = 8)

To simulate MRI‐guided cardiac radioablation, the XCAT data described above were modified using the digital CT/MRI breathing XCAT (CoMBAT) phantom for a balanced steady‐state free precession sequence with TR/TE = 10/5 ms.^97^, ^98^ Coronal and sagittal slice pairs were extracted from each 3D image to yield the desired 2D images. Volumes and slices had dimensions 128 × 128 × 128 and 128 × 128, respectively. Complex k‐space data were generated for each 2D image via Fourier transform.

#### 2.2.3 SPARE challenge (*n* = 2)

Imaging data for lung cancer radiation therapy were generated using scans from the sparse‐view reconstruction (SPARE) challenge.^99^ Briefly, 4D‐CT scans for patients with locally advanced nonsmall‐cell lung cancer receiving 3D conformal radiotherapy were acquired on two separate days. The scan for one day was used for training, while the scan for the other day was used for testing.

#### 2.2.4 Preprocessing

To generate training data, each 3D image of the 4D‐CT was forward‐projected at 680 imaging angles over one 360⁰ arc to yield a total of 6800 2D images as DRRs. To generate testing data, a real‐time position management (Varian Medical Systems, Palo Alto, US) trace for each patient was segmented into 10 respiratory bins. Then, for each imaging angle, the 3D image from the corresponding phase of the 4D‐CT was forward‐projected. For instance, if the first angle was 0⁰ and the first respiratory phase was bin 1, then the 3D image from bin 1 of the 4D‐CT was forward‐projected at 0⁰ to generate the first DRR, and so on until a total of 680 DRRs over one 360⁰ arc were produced.

All DRRs were generated for a 120 kVp beam with a pulse length of 20 ms going through a half‐fan bowtie filter. For the testing data, scatter and noise were generated at 40 mA tube current via Monte Carlo methods to simulate imaging conditions commonly encountered in radiotherapy. Unfortunately, of the nine patients originally included in the SPARE study, Monte Carlo simulations were only generated over two separate imaging days for two patients. In our previous work, we trained and tested Network A on data including scatter and noise.^88^ Here we validate Networks A‐C in the more realistic scenario where training occurs on data *without* scatter and noise, but testing occurs on data with scatter and noise.

Every 3D image was initially generated with voxel size 1 mm *×* 1 mm *×* 1 mm and dimensions 450 × 220 × 450 but was resized to 512 *×* 256 *×* 512 by cubic interpolation and downsampled to 128 *×* 128 *×* 128, yielding voxels of size 3.5 mm *×* 1.7 mm *×* 3.5 mm. Similarly, each DRR was initially generated with pixel sizes of 0.776 mm *×* 0.776 mm and dimensions 512 *×* 384, but was downsampled to 128 *×* 128 yielding pixels of size 3.1 mm × 2.3 mm.

Lung, rib, and planning target volume (PTV) masks were delineated by a clinician for each patient on the peak‐exhale 3D image. Lung masks were used to generate thoracoabdominal masks by generating a convex hull to include both lungs, expanding the resulting hull by binary dilation to include the ribs and extension inferiorly to the bottom of the image.

For the XCAT, CoMBAT, and SPARE data, every 3D image of the 4D‐CTs was registered to the peak‐exhalation 3D image using the Elastix toolkit^100^ to produce 3D DVFs. In particular, multiresolution registration was performed using B‐spline interpolation of order 3, optimizing for advanced Mattes Mutual Information with adaptive stochastic gradient descent and producing the resultant DVF via B‐spline transform. All DVFs were examined to ensure that there were no physiologically unrealistic registration results. All preprocessing steps were performed in MATLAB (The MathWorks, Inc., Natick, MA).

### 2.3 Training details

The *Voxelmap* architecture consists of an encoding arm(s), a decoding arm, integration layers, and a spatial transformation module. To describe the rationale behind each of these components: the encoding arm serves to produce a latent, low‐dimensional representation of the key features in the acquired and reference images, the decoding arm serves to map from this latent feature map to a 3D DVF, the integration layers encourage diffeomorphic transformations, and the spatial transformation module serves to deform the reference 3D image using the output 3D DVF to enable volumetric imaging.

The encoding arms consist of residual blocks where each input tensor is convolved with a kernel of size 4 *×* 4 with stride 2 and padding 1, followed by a kernel of size 3 *×* 3 with stride 1 and padding 1 and batch normalization. This repeating pattern of convolutions yields output tensors at half the dimension of the original inputs and the number of output channels is chosen as double that of the original input. In our preliminary work,^88^ we found that performance increased monotonically with network depth, hence the number of residual blocks is determined intrinsically by image size (e.g. the encoding arm of Network A has *n* = 7 residual blocks, since image size = 128 *×* 128 = 2^7^ *×* 2^7^) to yield a tensor with dimensions k *×* 1 *×* 1 or k *×* 1 *×* 1 *×* 1 (for 2D or 3D input images, respectively), where *k* is the number of channels.

With an equivalent number of residual blocks as the encoding arm, the decoding arm performs transpose convolution with a kernel of size 4 *×* 4 *×* 4 with stride 2 and padding 1, followed by a kernel of size 3 *×* 3 *×* 3 with stride 1 and padding 1 and batch normalization. Two additional convolutions are then performed at the desired output size with kernels of size 3 *×* 3 *×* 3 with stride 1 and padding 1, and the number of channels is chosen to produce a final output tensor (here with dimensions 3 *×* 128 *×* 128 *×* 128). Every convolution and transpose convolution uses ReLu activation, except the penultimate and final layers which use Tanh and linear activation, respectively.

The output of the decoding arm is then fed through *scaling and squaring layers*, which efficiently integrate the corresponding tensor. In particular, since $\varphi^{\left(1\right)}=e^{\left(u\right)}$ is a solution to^1^ and $e^{(a+b)u}=e^{au}\;\cdot \;e^{bu}$ for a,b∈R, we use the recurrence $\varphi^{\left(1\;/\;2^{T-1}\right)}=\varphi^{\left(1\;/\;2^{T}\right)}\cdot \;\varphi^{\left(1\;/\;2^{T}\right)}$ to obtain $\varphi^{\left(1\right)}=\varphi^{\left(1\;/\;2\right)}\cdot \;\varphi^{\left(1\;/\;2\right)}$ by choosing T large enough, such that stationary velocity field u≈0 ^86^, ^87^ (here we choose T=10). Following this integration procedure, the resulting 3D DVF can then be fed into a spatial transform module along with a reference 3D image to produce a predicted 3D image.

All neural networks were trained by minimizing $MSE(\varphi ,\hat{\varphi })$ within a thoracoabdominal mask using the Adam learning algorithm^101^ with learning rate 1 × 10^−5^ and batch size 8 for 50 epochs on a NVIDIA RTX A6000 48 GB GPU, requiring 6–8 h. Deep learning experiments were performed in the Pytorch machine learning framework.^102^

### 2.4 Evaluation

Although a wide array of active motion management techniques exist, such as tracking and gating,^103^ a passive approach is typically employed. In particular, margin expansion via an internal target volume (ITV) or probabilistic margins both aim to encompass tumor position in the presence of intrafraction motion.^104^, ^105^ However, these approaches may result in large doses delivered to OARs^106^, ^107^, ^108^ and do not guarantee target coverage, especially in cases of tumor drift. Given the absence of an agreed‐upon gold‐standard for real‐time 3D motion estimation and volumetric imaging, as highlighted in a recent critical review,^109^ and considering that specialized tracking systems represent a different class of technology than standard radiotherapy systems, we establish a clinically‐relevant baseline by comparing the performance of *Voxelmap* to the current standard‐of‐care ITV‐based approach. That is, the target and OAR volumes are defined as those which encompass the positions of these structures over the entire respiratory cycle. Target volumes were recorded for all patients and phantoms. (Note: conventionally, the ITV is defined as the gross target volume (GTV) envelope plus the envelope from the 4D scan. Here we define the ITV as the integral volume traversed by the target in the 4D scan from the beam frame of reference. For the standard‐of‐care, the beam remains static. Using *Voxelmap*, the beam is dynamically shifted based on predicted 3D motion)

To measure geometric accuracy, we used Dice similarity (DSC):

(2) $$DSC=\frac{1}{T}\sum_{t=1}^{T}\frac{2\;\left|G_{t}\;\cap \;P_{t}\right|}{\left|G_{t}\right|+\left|P_{t}\right|}$$

where $G_{t}$ and $P_{t}$ are the set of points in the ground‐truth and predicted contour, respectively, at time t.

For the targets, we also computed the centroid error (CE), defined as the Euclidean distance between the centroids of the predicted and ground‐truth segmentations at time t:

(3) $$CE\left(t\right)={\left|\left|\;\mu_{P\left(t\right)}\;-\;\mu_{G\left(t\right)}\;\right|\right|}_{2}$$

where $\mu_{P(t)}$ and $\mu_{G(t)}$ are the centre‐of‐mass spatial coordinates of the predicted and ground‐truth target segmentations, respectively.

To assess image accuracy, we computed the structural similarity (SSIM):

(4) $$SSIM=\frac{\left(2\mu_{x}\mu_{y}+c_{1}\right)\left(2\sigma_{xy}+c_{2}\right)}{\left(\mu_{x}^{2}+\mu_{y}^{2}+c_{1}\right)\left(\sigma_{x}^{2}+\sigma_{y}^{2}+c_{1}\right)}$$

where x and y are ground‐truth and predicted 3D images, respectively, while $c_{1}$ and $c_{2}$ are variables used to stabilize division in cases of weak denominators.

To examine the diffeomorphic properties of predicted DVFs, we used the Jacobian determinant (detJ):

(5) $$detJ=\left|\nabla \hat{\varphi_{t}}\left(v\right)\right|$$

where $\nabla \hat{\varphi_{t}}\left(v\right)$ is the matrix of first‐order partial derivatives of the predicted DVF $\hat{\varphi_{t}}$ at time t in the local neighborhood around voxel v and |·| is the determinant. Since $\hat{\varphi_{t}}$ is topology‐preserving and invertible only where detJ>0, we recorded the proportion of voxels for which this condition is violated.^85^, ^86^

## 3 RESULTS

### 3.1 XCAT experiments

**FIGURE 3:**
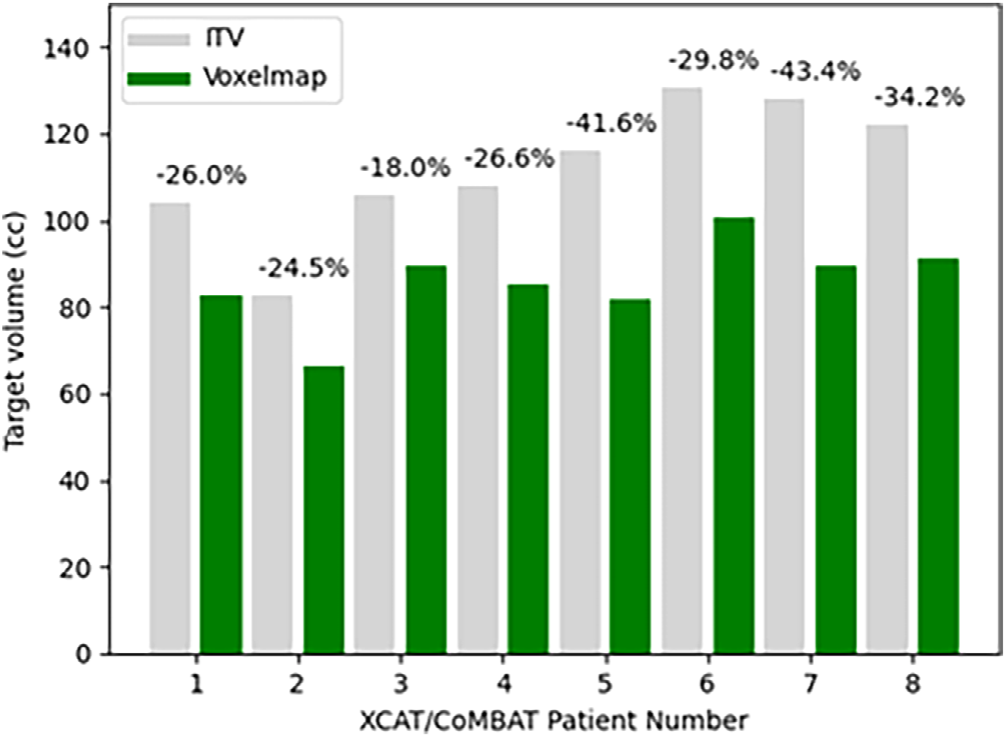
Bar plot of target volumes comparing an ITV‐based approach (in grey) to *Voxelmap* (in green) for the XCAT/CoMBAT phatoms. The percent difference in target volume is shown above the bar pairs.

**FIGURE 4:**
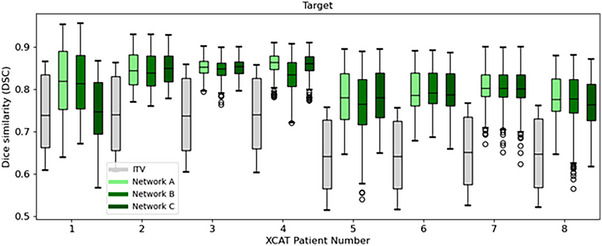
Box plot comparing Dice similarity of the target for an ITV‐based approach (in grey) to Networks A (in light green), B (in green), and C (in dark green) for the XCAT phantoms.

The performance of *Voxelmap* on the XCAT x‐ray data was evaluated by training Networks A, B, and C in a patient‐specific manner. As shown in Figure 3, target volumes that were dynamically shifted using *Voxelmap* were on average 30.5 % smaller than those using an ITV‐based approach. As shown in Figure 4, *Voxelmap* consistently outperformed the ITV‐based approach in terms target position with a mean DSC across all phantoms of 0.70 ± 0.08, 0.82 ± 0.05, 0.81 ± 0.05, and 0.82 ± 0.05 for ITV and Networks A, B, and C, respectively.

**FIGURE 5:**
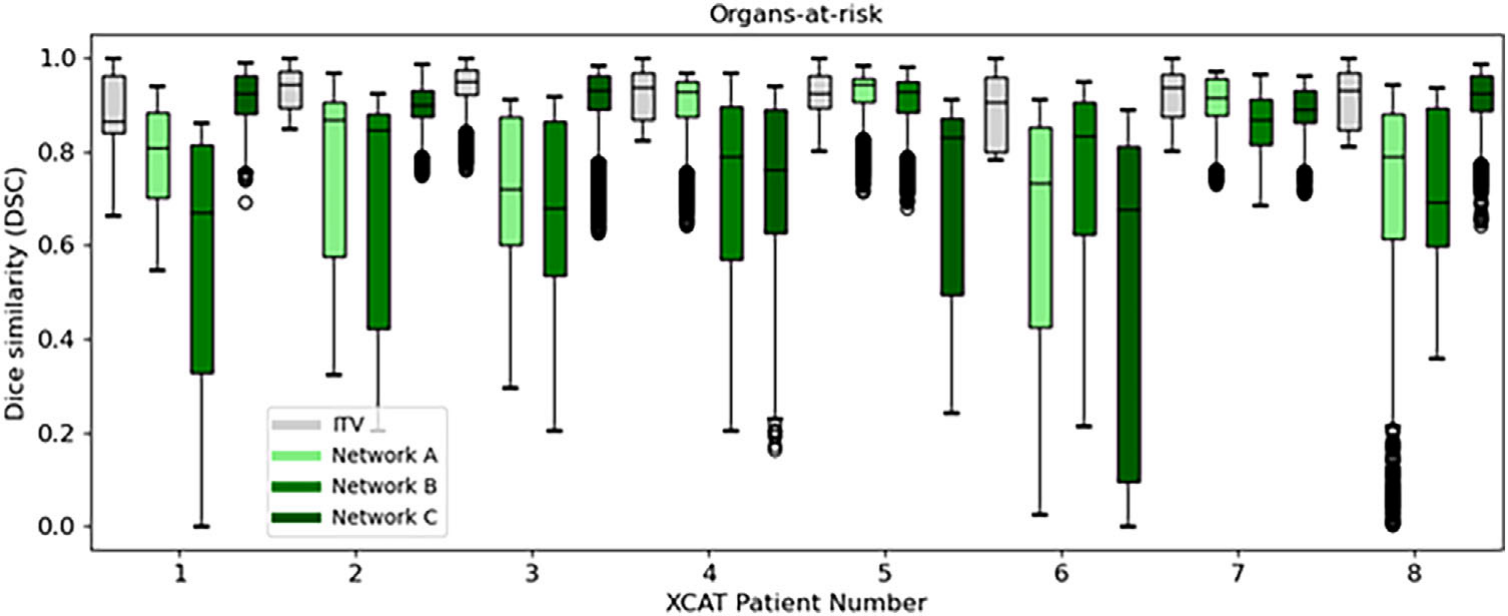
Box plot comparing Dice similarity of the organs‐at‐risk for an ITV‐based approach (in grey) to Networks A (in light green), B (in green), and C (in dark green) for the XCAT phantoms. Here, for the sake of brevity, we show the performance averaged over all organs‐at‐risk (i.e. the stomach, esophagus, left lung, right lung, and spinal cord).

**FIGURE 6:**
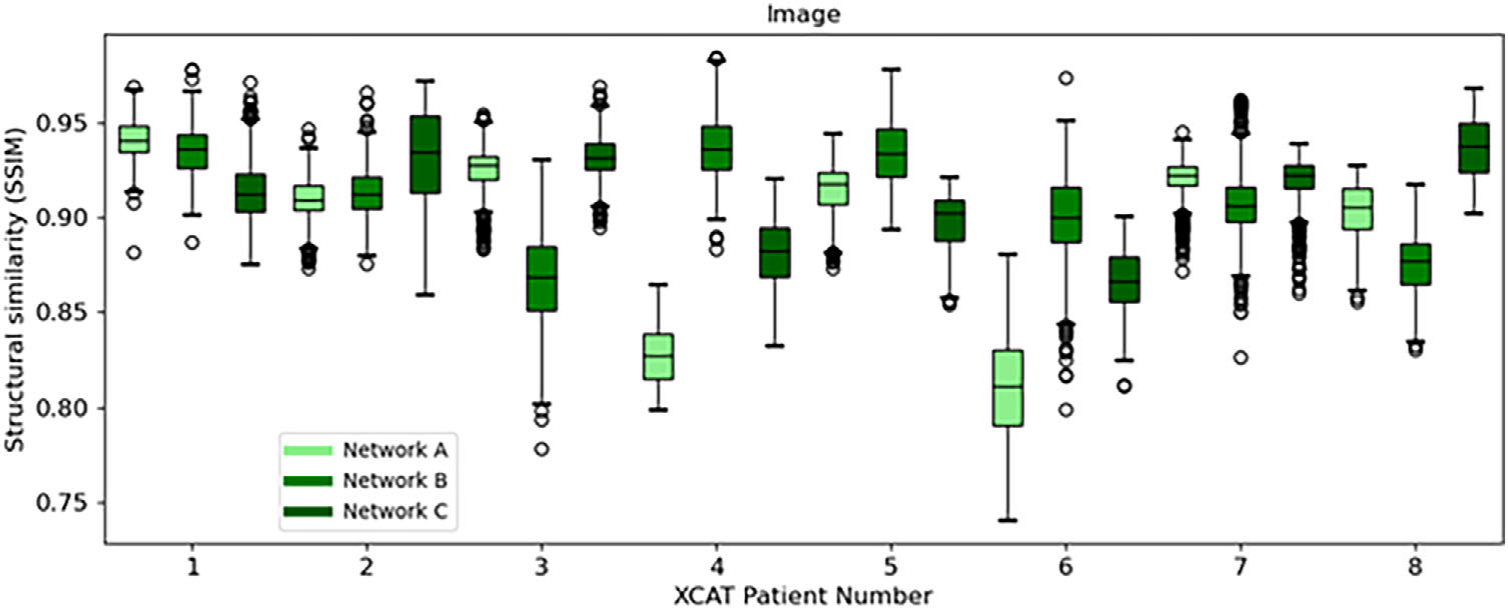
Box plot comparing structural similarity of predicted volumetric images for Networks A (in light green), B (in green), and C (in dark green) for the XCAT phantoms.

As shown in Figure 5, the ITV‐based approach outperformed *Voxelmap* in terms of organ‐at‐risk position with a mean DSC of 0.92 ± 0.01, 0.79 ± 0.05, 0.78 ± 0.04, and 0.81 ± 0.04 for ITV and Networks A, B, and C, respectively (see Table S1 for more details). As shown in Figure 6, in terms of image similarity, Networks A, B, and C performed comparably with a mean SSIM of 0.89 ± 0.01, 0.90 ± 0.02, and 0.91 ± 0.02. All networks produced diffeomorphic mappings, as detJ>0 for every voxel of every predicted DVF.

**FIGURE 7:**
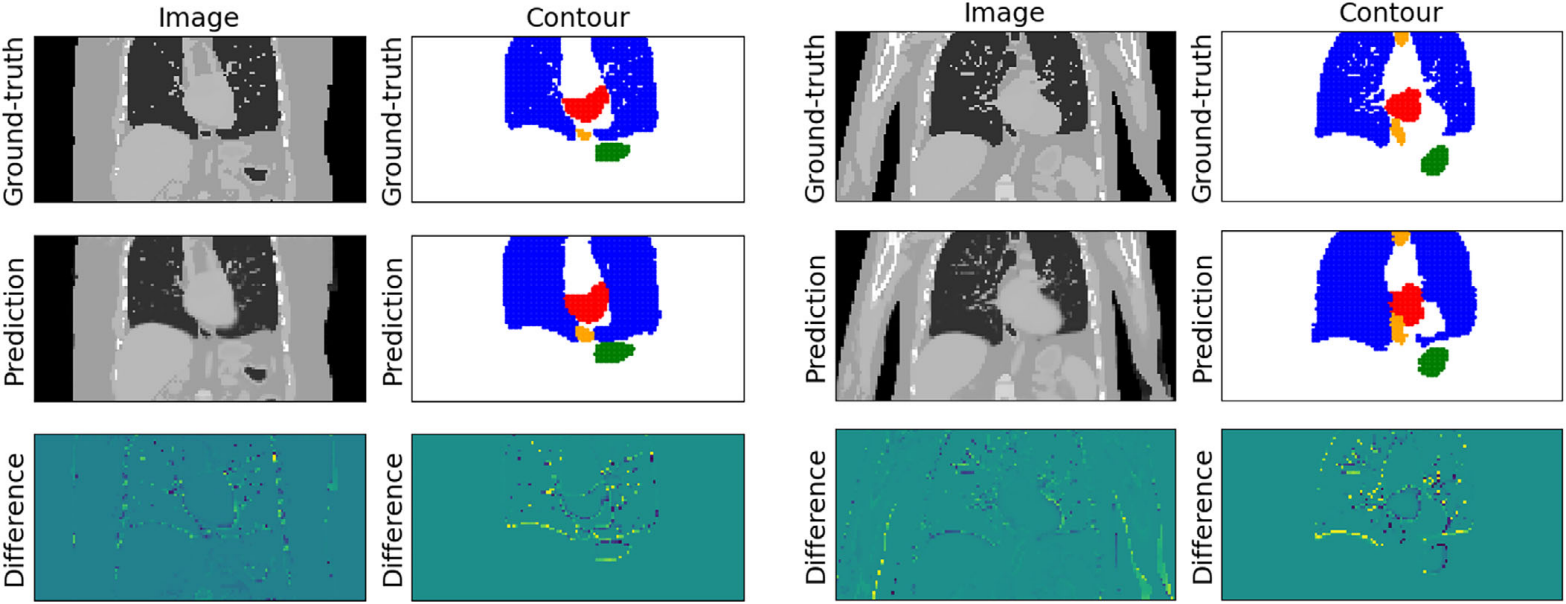
Example images, contours, and difference maps for the best‐performing (Patient 4, Network A, left) and worst‐performing XCAT case (Patient 5, Network B, right). Contours for the target, stomach, esophagus, lungs, and spinal cord are shown in red, green, orange, blue, and pink, respectively.

In terms of target position, the best performing XCAT case was Patient 4 using Network A in which a mean DSC of 0.86 was observed; conversely, the worst performing XCAT case was Patient 5 using Network B in which a mean DSC of 0.77 was observed (Figure 7). This difference in performance is likely due to the fact that Phantoms 1–4 were programmed with diaphragm motion half that of Phantoms 5–8.

As shown in Table 1, *Voxelmap* consistently outperformed the ITV‐based method in terms of target centroid error. The overall mean across phantoms was 3.2 mm for ITV, compared to 2.0, 2.3, and 2.3 mm for Networks A, B, and C, respectively.

**TABLE 1:** Centroid error (in mm) for the XCAT data.

Phantoms	ITV	Network A	Network B	Network C
1	2.0 ± 1.1	1.4 ± 0.7	1.5 ± 0.7	1.6 ± 0.7
2	2.1 ± 1.3	1.5 ± 0.8	1.4 ± 0.8	1.6 ± 1.0
3	2.3 ± 1.3	1.4 ± 0.9	2.2 ± 1.2	1.4 ± 0.8
4	2.1 ± 1.3	2.0 ± 1.3	1.6 ± 0.8	1.6 ± 0.9
5	4.0 ± 2.4	2.5 ± 1.6	3.5 ± 2.2	2.7 ± 1.7
6	4.6 ± 2.6	2.4 ± 1.6	2.7 ± 1.6	4.4 ± 2.5
7	4.0 ± 2.1	2.4 ± 1.2	3.0 ± 1.7	2.3 ± 1.3
8	4.2 ± 2.4	2.6 ± 1.3	2.3 ± 1.5	2.7 ± 1.4
**Overall**	**3.2 ± 1.1**	**2.0 ± 0.5**	**2.3 ± 0.7**	**2.3 ± 0.9**

### 3.2 CoMBAT experiments

**FIGURE 8:**
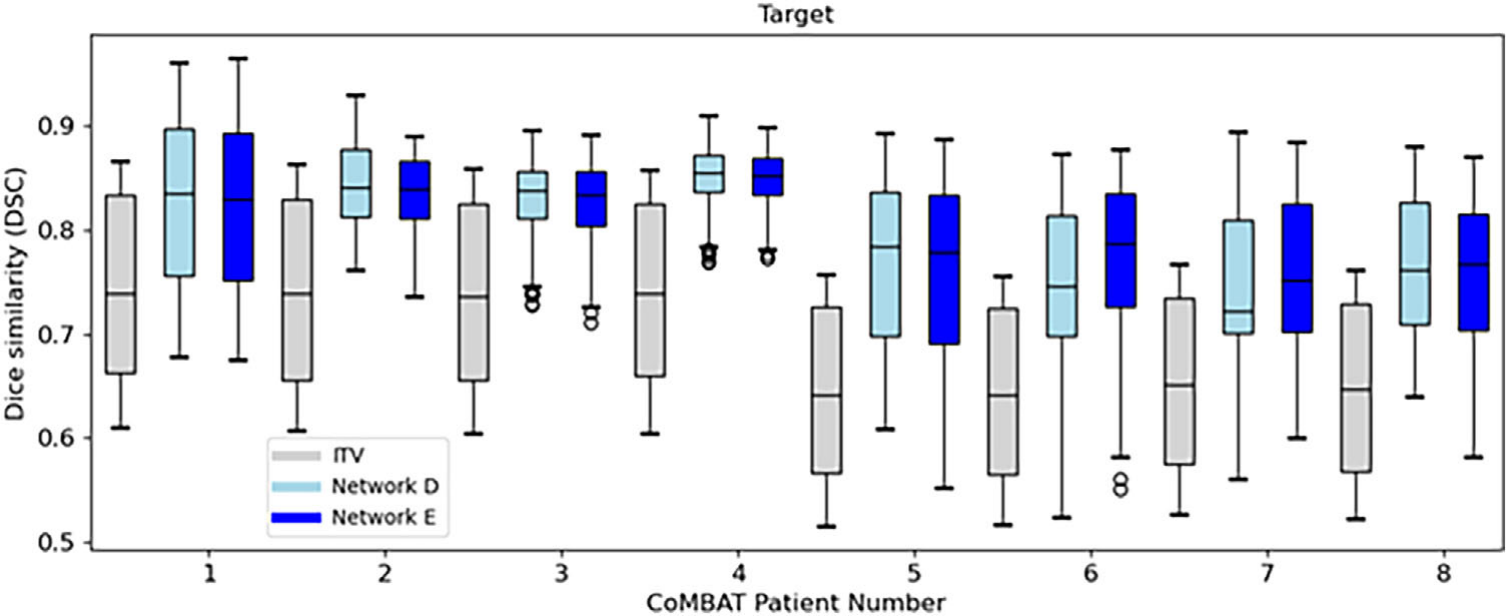
Box plot comparing Dice similarity of the target for an ITV‐based approach (in grey) to Networks D (in light blue), and E (in blue) for the CoMBAT phantoms.

The performance of *Voxelmap* on the CoMBAT MRI data was evaluated by training Networks D and E in a patient‐specific manner. (Since the XCAT data were used to generate the CoMBAT data, the same 30.5 % reduction in target volume size can be attributed to these experiments as well). Although training data could be generated abundantly for x‐ray guided treatments via forward‐projection, there is a dearth of training data for MRI guided treatments if only the central coronal‐sagittal slices of the pretreatment 4D‐MRI are used. Therefore, off‐center slices were also used. Here, Networks D and E were trained on all phantom data using the central 16 × 16 slice pairs (see Figure S‐1 for details). As shown in Figure 8, *Voxelmap* consistently outperformed the ITV‐based approach in terms target position with a mean DSC across all phantoms of 0.70 ± 0.08, 0.80 ± 0.06, and 0.80 ± 0.06 for ITV and Networks D, and E, respectively.

**FIGURE 9:**
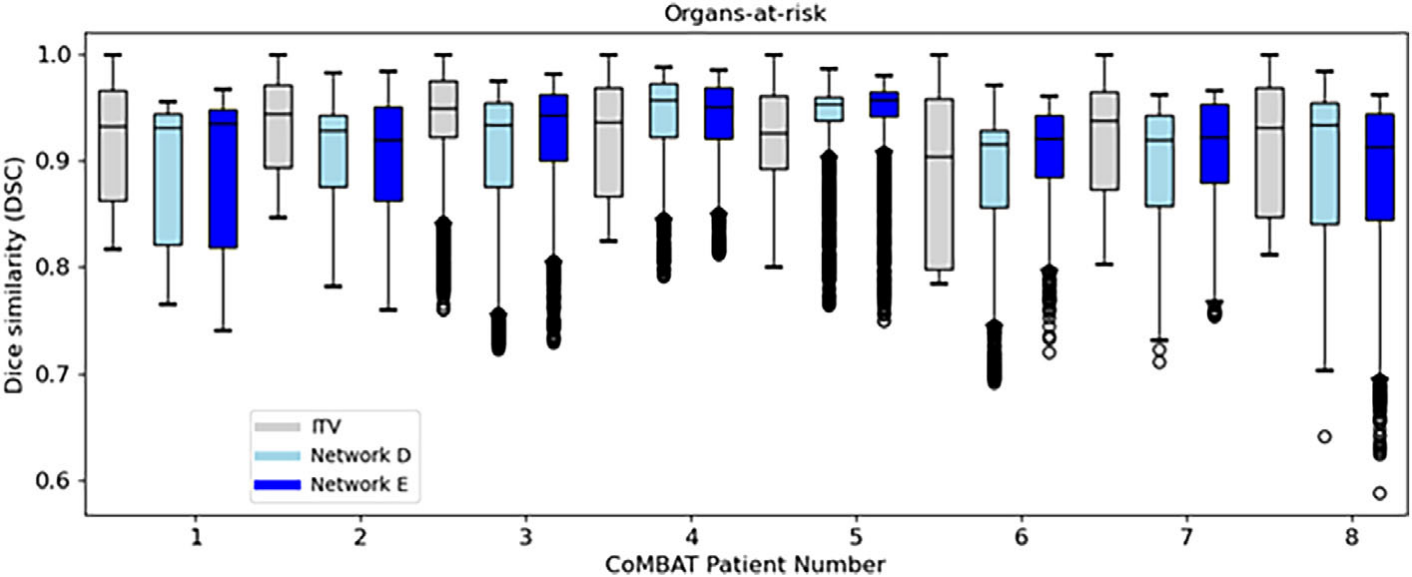
Box plot comparing Dice similarity of the organs‐at‐risk for an ITV‐based approach (in grey) to Networks D (in light blue), and E (in blue) for the CoMBAT phantoms. Here, for the sake of brevity, we show the performance averaged over all organs‐at‐risk (i.e. the stomach, esophagus, left lung, right lung, and spinal cord).

**FIGURE 10:**
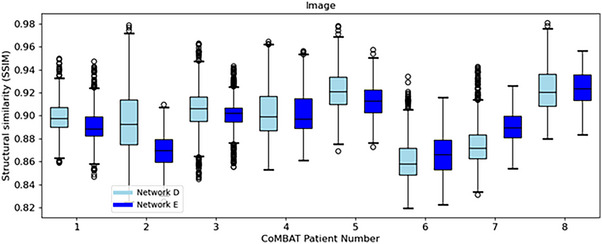
Box plot comparing structural similarity of predicted volumetric images for Networks D (in light blue), and E (in blue) for the CoMBAT phantoms.

As shown in Figure 9, *Voxelmap* performed comparably to an ITV‐based approach in terms of organ‐at‐risk position with a mean DSC of 0.92 ± 0.01, 0.91 ± 0.02, and 0.91 ± 0.02 for ITV and Networks D, and E, respectively (see Table S2 for more details). As shown in Figure 10, in terms of image similarity, Networks D and E performed equivalently with a mean SSIM of 0.90 ± 0.02. All networks produced diffeomorphic mappings, as detJ>0 for every voxel of every predicted DVF.

**FIGURE 11:**
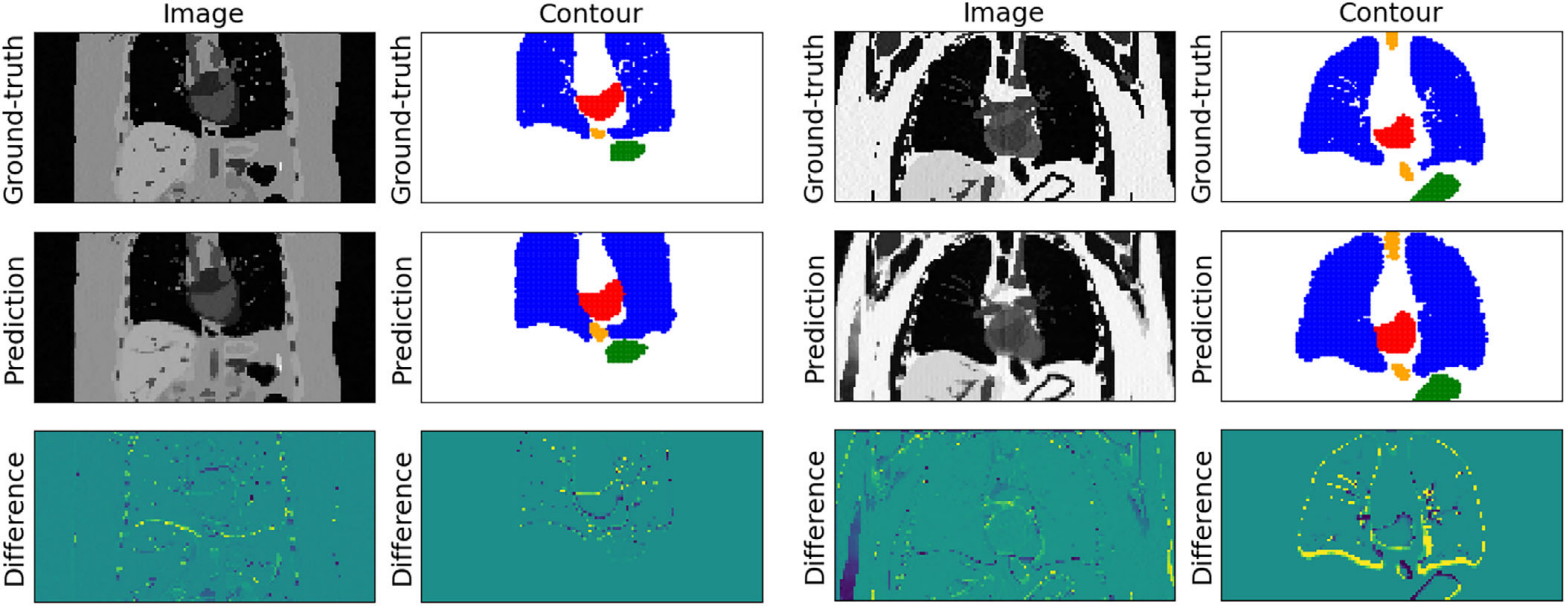
Example images, contours, and difference maps for the best‐performing (Patient 4, Network D, left) and the worst‐performing (Patient 7, Network D, and right) CoMBAT case. Contours for the target, stomach, esophagus, lungs, and spinal cord are shown in red, green, orange, blue, and pink, respectively.

In terms of target position, the best‐performing CoMBAT case was Patient 4 using Network D in which a mean DSC of 0.85 was observed; conversely, the worst‐performing CoMBAT case was Patient 7 also using Network D in which a mean DSC of 0.74 was observed (Figure 11).

As shown in Table 2, *Voxelmap* consistently outperformed the ITV‐based method in terms of target centroid error. The overall mean across phantoms was 3.2 mm for ITV, compared to 1.7 mm, and 1.8 mm for Networks D, and E, respectively.

**TABLE 2:** Centroid error (in mm) for the CoMBAT data.

Phantoms	ITV	Network D	Network E
1	2.0 ± 1.1	0.9 ± 0.4	1.0 ± 0.4
2	2.1 ± 1.3	0.8 ± 0.5	0.9 ± 0.6
3	2.3 ± 1.3	1.2 ± 0.7	1.5 ± 0.8
4	2.1 ± 1.3	0.8 ± 0.4	0.9 ± 0.4
5	4.0 ± 2.4	2.4 ± 1.5	2.6 ± 1.6
6	4.6 ± 2.6	1.9 ± 1.1	2.2 ± 1.4
7	4.0 ± 2.1	2.8 ± 1.4	2.7 ± 1.4
8	4.2 ± 2.4	2.4 ± 1.4	2.6 ± 1.4
**Overall**	**3.2 ± 1.1**	**1.7 ± 0.8**	**1.8 ± 0.8**

### 3.3 SPARE experiments

**FIGURE 12:**
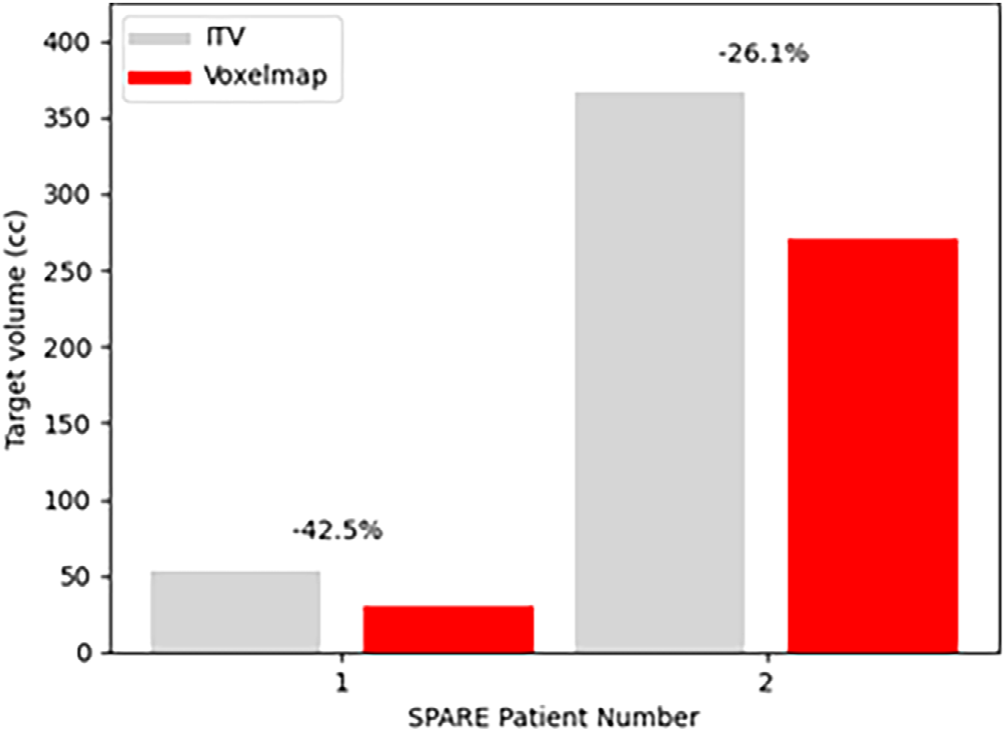
Bar plot of target volumes comparing an ITV‐based approach (in grey) to *Voxelmap* (in red) for the SPARE patients. The percent difference in target volume is shown above the bar pairs.

**FIGURE 13:**
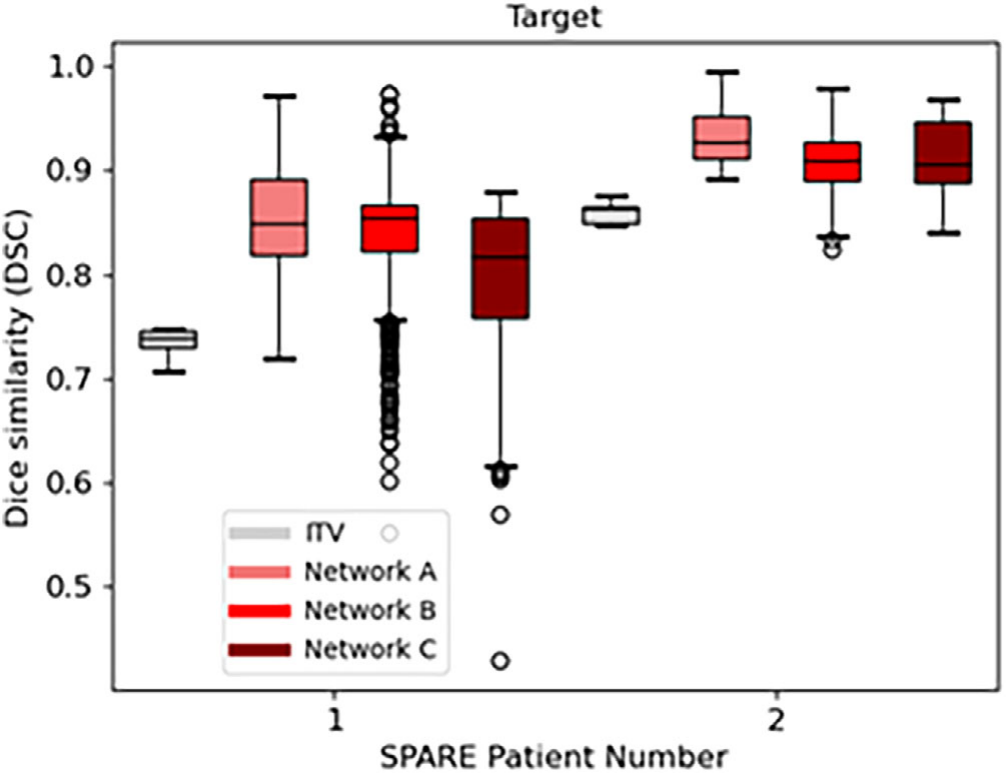
Box plot comparing Dice similarity of the target for an ITV‐based approach (in grey) to Networks A (in light red), B (in red), and C (in dark red) for the SPARE patients.

The performance of *Voxelmap* on the SPARE patient data was evaluated by training Networks A, B, and C in a patient‐specific manner. As shown in Figure 12, target volumes that were dynamically shifted using *Voxelmap* were on average 34.8 % smaller than those using an ITV‐based approach. As shown in Figure 13, *Voxelmap* consistently outperformed the ITV‐based approach in terms target position with a mean DSC across all patients of 0.80 ± 0.01, 0.89 ± 0.04, 0.88 ± 0.04, and 0.86 ± 0.05 for ITV and Networks A, B, and C, respectively.

**FIGURE 14:**
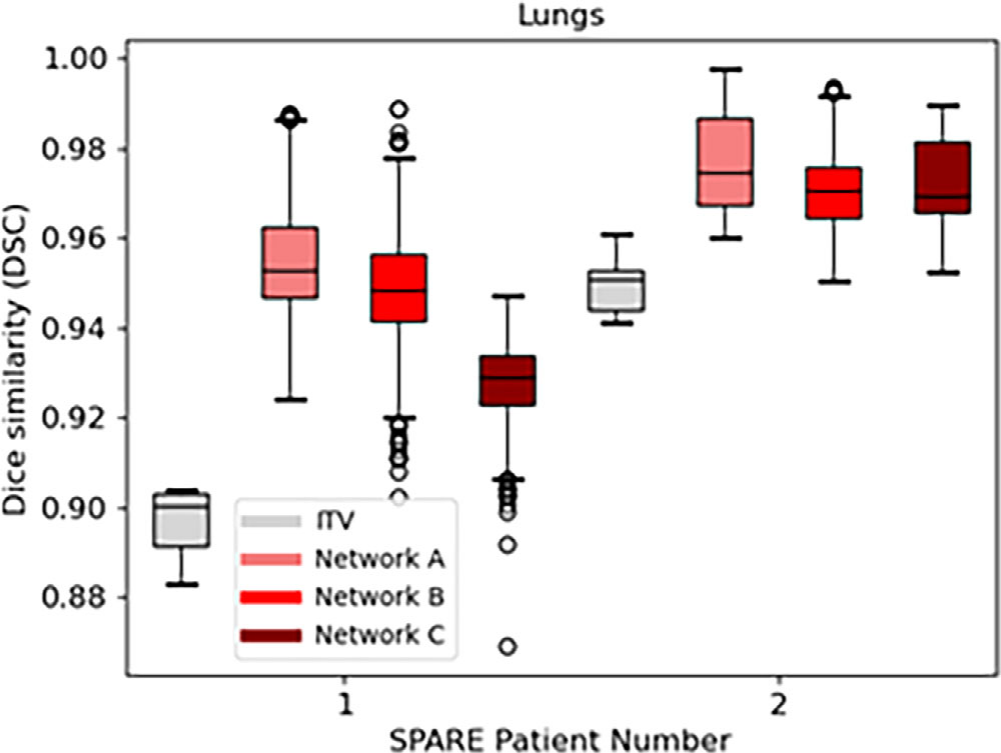
Box plot comparing Dice similarity of the lungs for an ITV‐based approach (in grey) to Networks A (in light red), B (in red), and C (in dark red) for the SPARE patients.

**FIGURE 15:**
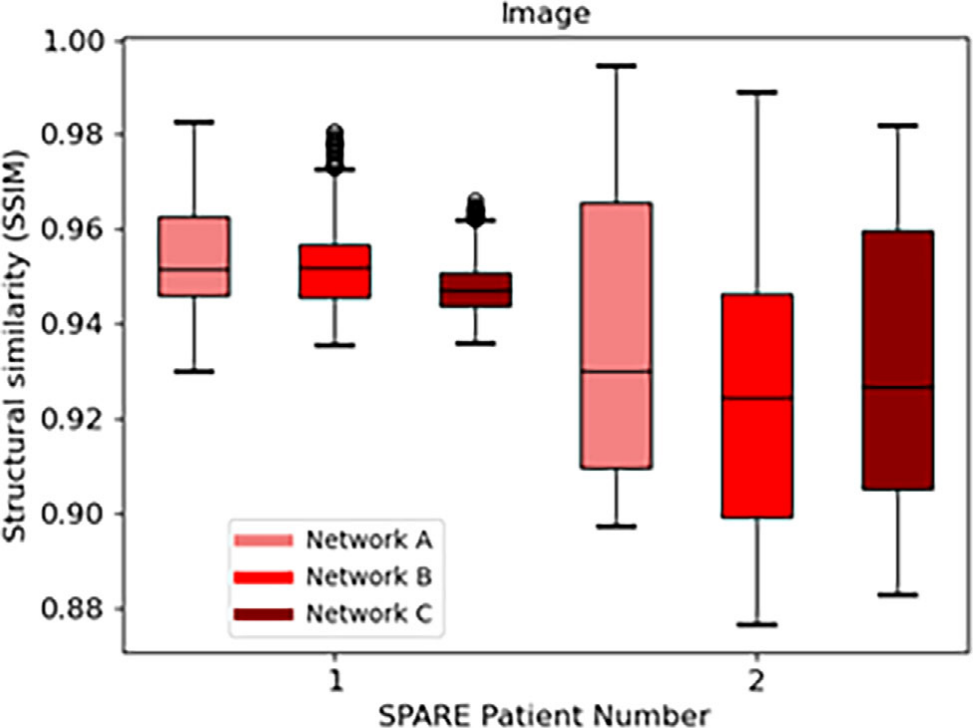
Box plot comparing structural similarity of predicted volumetric images for Networks A (in light red), B (in red), and C (in dark red) for the SPARE patients.

Additionally, as shown in Figure 14, *Voxelmap* also outperformed the ITV‐based approach in terms of lung position with a mean DSC of 0.93 ± 0.01, 0.97 ± 0.01, 0.96 ± 0.01, and 0.94 ± 0.01 for ITV and Networks A, B, and C, respectively. As shown in Figure 15, in terms of image similarity, Networks A, B, and C performed comparably with a mean SSIM 0.95 ± 0.02, 0.94 ± 0.02, and 0.94 ± 0.02. All networks produced diffeomorphic mappings, as detJ>0 for every voxel of every predicted DVF.

As shown in Table 3, Network A consistently outperformed the ITV‐based method in terms of target centroid error with overall mean across patients of 2.3 mm for ITV, compared to 1.8 mm. However, Networks B and C underperformed relative to an ITV‐based approach with mean centroid errors of 2.7 and 3.5 mm, respectively.

**TABLE 3:** Centroid error (in mm) for the SPARE data.

Patient	ITV	Network A	Network B	Network C
1	2.5 ± 1.1	2.0 ± 0.7	2.9 ± 1.1	4.0 ± 1.5
2	2.2 ± 0.8	1.7 ± 0.7	2.5 ± 1.2	3.0 ± 1.2
Overall	2.3 ± 1.0	1.8 ± 0.7	2.7 ± 1.2	3.5 ± 1.4

## 4 DISCUSSION

### 4.1 Generalizability

We propose *Voxelmap*, an open‐source deep learning framework for intrafraction respiratory motion estimation and volumetric imaging that can be flexibly deployed across image modalities. In terms of target position, we found relatively similar performance on the XCAT x‐ray and CoMBAT MRI data with mean DSC of 0.82 and 0.80, respectively. However, with the improved soft‐tissue contrast, we observed better performance on the MRI data in predicting OAR position with a mean DSC of 0.90 as compared to 0.79 on the x‐ray data. In fact, the ITV‐based approach outperformed *Voxelmap* on the XCAT data but, it should be noted that these data represent a unique scenario without interfraction changes and where the positions of every structure are known exactly. Indeed, on the SPARE x‐ray data we found that *Voxelmap* outperformed the ITV‐based approach with a mean DSC of 0.96 and 0.93, respectively. In terms of target position, *Voxelmap* exhibited mean centroid errors of 2.2 and 1.8 mm on the XCAT and CoMBAT data, respectively, placing performance well within the suggested 3–5 mm margins for cardiac radioablation.^110^ For the SPARE data, while Network A showed consistent and improved centroid accuracy over the ITV baseline (1.8 and 2.3 mm, respectively), Networks B and C did not follow this trend (2.7 and 3.5 mm, respectively). Importantly, while the XCAT data were “clean”, the SPARE testing data contained scatter and noise typical of that encountered in clinical settings. Therefore, to assess the impact of noise, we performed feature correlation analysis (see Table S3 for more details) which revealed that Network A exhibited high representational stability across noise conditions, while B and C exhibited more volatile behavior that only stabilized at high noise levels. This aligns with the architectural differences: Network A fuses acquired and reference 2D images early, enabling the encoder to learn noise‐robust, jointly‐informative features. In contrast, Network B encodes acquired and reference images separately, which may introduce inconsistencies between learned representations, especially in the presence of mild noise or ambiguous motion cues. Similarly, the use of full 3D information in Network C alongside 2D data may introduce modality mismatch, complicating feature fusion during decoding. These observations suggest that early fusion architectures like Network A may be more resilient to the kinds of image noise and anatomical ambiguity present in real clinical settings. Nonetheless, *Voxelmap* performance on the SPARE data is also within suggested clinical tolerances for stereotactic lung treatments.^111^

One important observation is similar performance in taking MR images and k‐space data in Networks D and E, respectively. However, it should be noted that Network E incorporated 18% more trainable parameters. Moreover, while future work will look to validate the method on data from physical instruments, the k‐space data were simulated directly from MR images via Fourier transform. Nonetheless, we sought to demonstrate proof‐of‐principle for real‐time motion estimation and volumetric imaging with raw k‐space data as it could significantly improve system latency for MRI guidance because; first, online image reconstruction can be averted and, second, Zhang et al. have demonstrated dramatic speed and memory improvements by performing image registration tasks in a bandlimited, low‐frequency space with negligible impact on accuracy.^112^, ^113^, ^114^ Neural networks have also been used to achieve real‐time image reconstruction by enforcing data consistency with k‐space,^115^ which could offer a promising alternative approach.

One limitation is that while the x‐ray‐based networks were validated on both synthetic and patient data (XCAT and SPARE, respectively), the MRI‐based networks were only validated on the synthetic CoMBAT data. These experiments reflect the uniqueness of the SPARE data in supplying patient data with ground‐truth volumetric images. To our knowledge, there is no equivalent dataset in the context of MRI‐guided radiation therapy.

### 4.2 Interfraction variation

**FIGURE 16:**
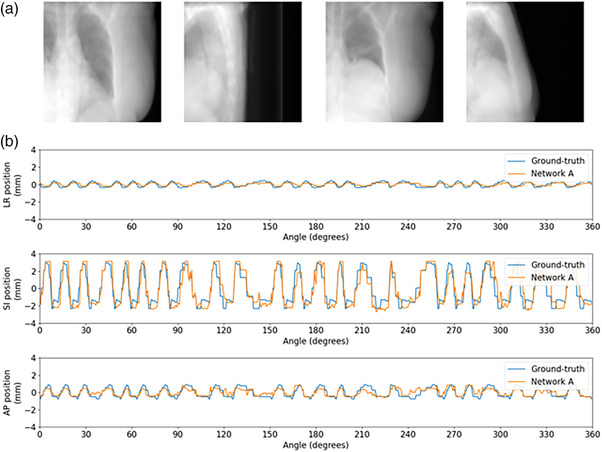
Example tumor tracking plot for SPARE patient 2. (a) From left to right the panels show projections at 0⁰, 90⁰, 180⁰, and 270⁰. (b) From top to bottom the panels show motion traces along the left‐right (LR), superior‐inferior (SI), and anterior‐posterior (AP) axes with the ground‐truth and Network A prediction in blue and orange, respectively.

**FIGURE 17:**
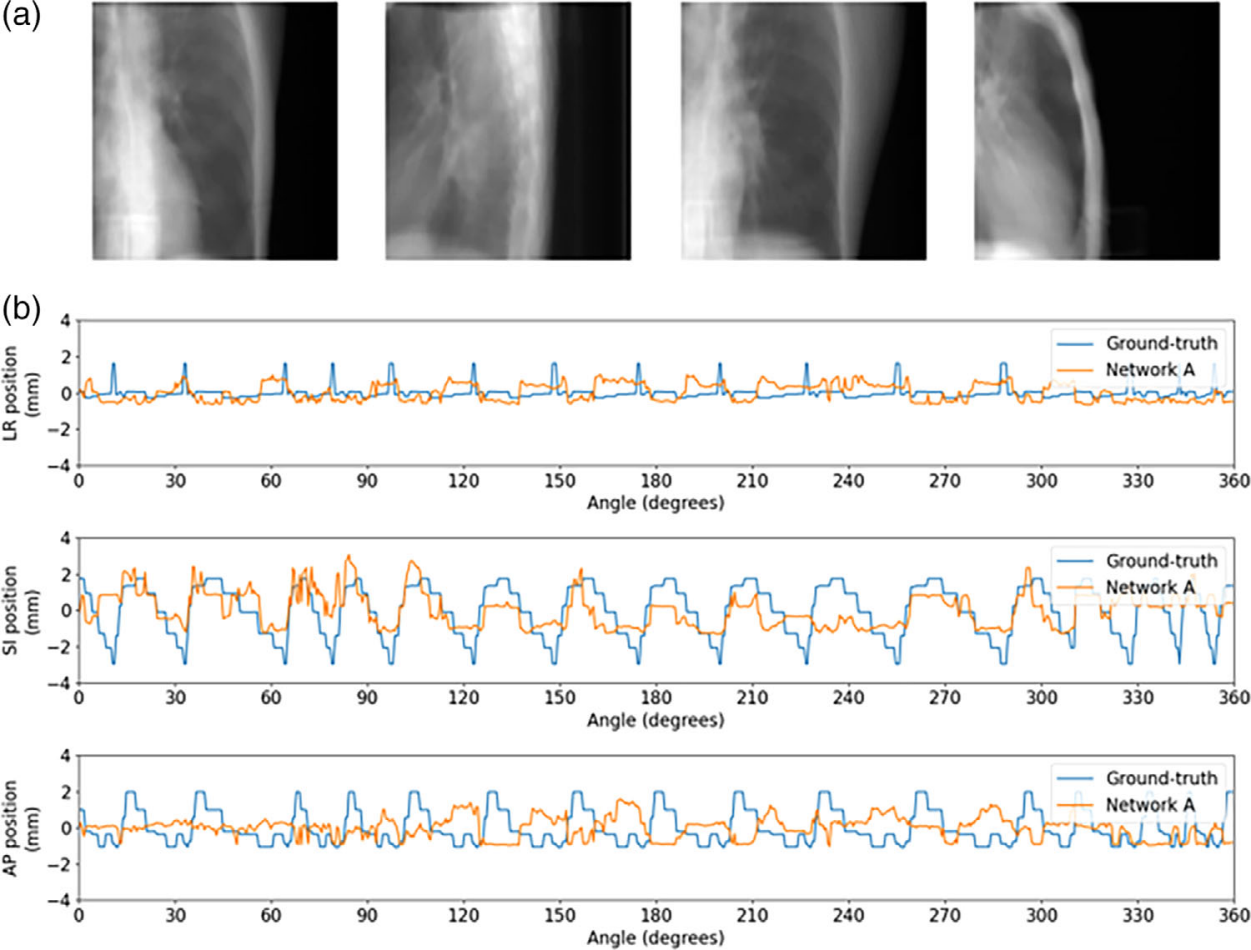
Example tumor tracking plot for SPARE patient 1. (a) From left to right the panels show projections at 0⁰, 90⁰, 180⁰, and 270⁰. (b) From top to bottom the panels show motion traces along the left‐right (LR), superior‐inferior (SI), and anterior‐posterior (AP) axes with the ground‐truth and Network A prediction in blue, and orange, respectively.

As presented here, *Voxelmap* is trained on a 4D scan consisting of 10 volumes, which represents a very limited sample of the wide range of possible deformations that may occur between breathing cycles and between treatment days. To overcome this challenge, Jiang et al. proposed a data augmentation strategy to generate synthetic deformations, translations, and rotations.^116^ However, these deformations were created by randomly sampling the eigenvalues of principal components derived from 4D imaging. Therefore, while this method improved the robustness of their method, these deformations nonetheless reflect linear perturbations of one breathing cycle. Moreover, these deformations, translations, and rotations were generated within hand‐crafted ranges to ensure physiologically realistic results. Another possibility is to use test‐time adaptation,^117^ by using neural networks to find an approximate distribution of “feasible” deformations, which is then sampled. It should be noted that, for the SPARE data, *Voxelmap* was trained and tested on 4D‐CTs acquired on separate days and we found excellent agreement between, for instance, the predicted and ground‐truth centroid positions of the target for Patient 2 (Figure 16). Unfortunately, we observed a degradation in performance for Patient 1; however, we hypothesize that this occurred because the diaphragm was not persistently visible (Figure 17). These results suggest that, in the case of a standard linear accelerator, an extended field‐of‐view may be required if *Voxelmap* is to be used for motion adaptive treatments. However, given that this method could result in using treatment volumes that are >30% smaller, we suggest that the added kV dose will be negligible compared to the spared MV dose.

### 4.3 Learned representations

**FIGURE 18:**
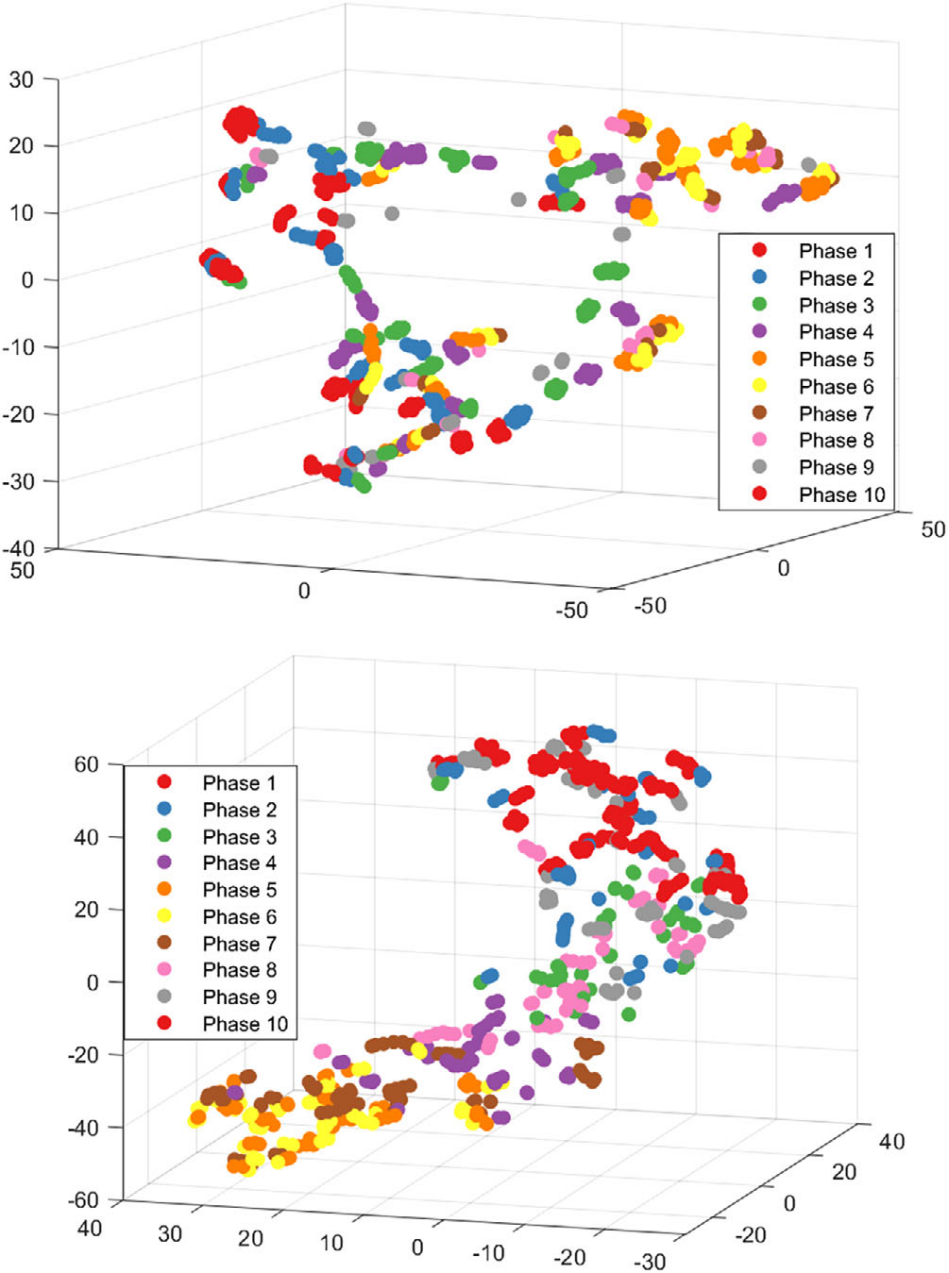
3D scatter plot of t‐SNE results of the low‐dimensional feature map of Network A for SPARE patient 1 (top) and 2 (bottom). Each point corresponds to the output activations of the encoding arm for an acquired projection and is labelled according to its phase in the respiratory cycle.

A key idea for our proposed framework is that the solution space for 3D internal organ motion is constrained by the anatomy and biomechanics of the patient. Mathematically, we suggest that this solution space forms a *manifold* (that is, a locally Euclidean topological object) and that our learning task can be formulated as one of manifold approximation. To illustrate this notion, we used t‐distributed stochastic neighbor embedding (t‐SNE),^118^ to visualize the activations of the final encoding block of Network A across the SPARE testing data. As shown in Figure 18, the t‐SNE representation for Patient 1 has a torus‐like structure, in which inhale and exhale points span the entire surface in a cyclical manner. We hypothesize that this structure arose because the diaphragm was not persistently visible; hence, the network did not have access to a reliable surrogate and was unable to anticipate sudden changes in position. Conversely, for Patient 2, where the diaphragm was persistently visible, the t‐SNE representation formed a smooth surface in which intermediate phases connect disjoint inhale and exhale clusters. Notwithstanding these differences in learned representations, *Voxelmap* had similar performance in terms of target and lung overlap across both patients.

### 4.4 Nonrespiratory motion

**FIGURE 19:**
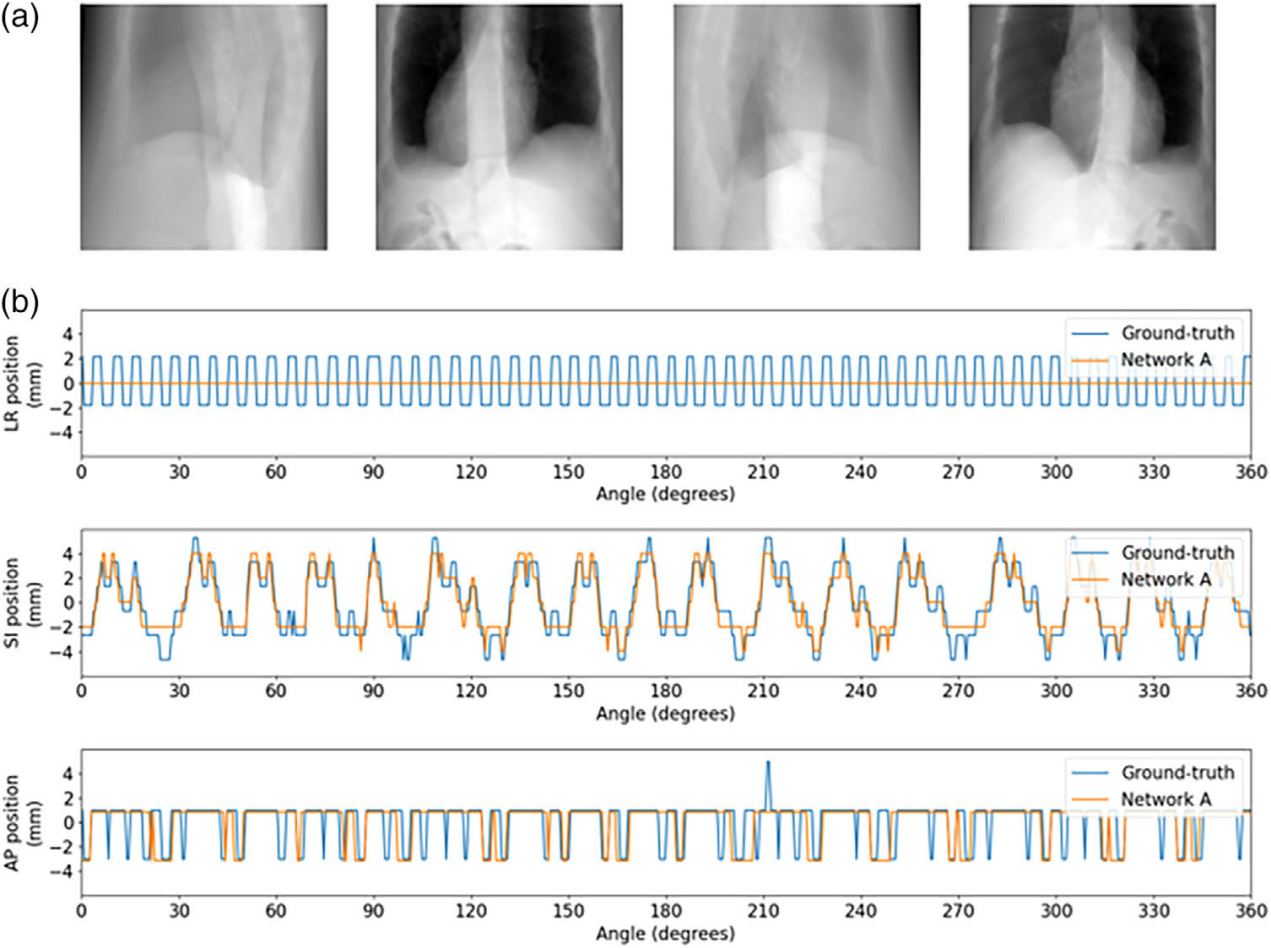
Example target tracking plot for XCAT patient 1. (a) From left to right the panels show projections at 0⁰, 90⁰, 180⁰, and 270⁰. (b) From top to bottom the panels show motion traces along the left‐right (LR), superior‐inferior (SI), and anterior‐posterior (AP) axes with the ground‐truth and Network A prediction in blue, and orange, respectively.

Since only respiratory motion will be observed on the 4D scan, cardiac‐induced deformation will not be captured by *Voxelmap*. From a manifold‐learning perspective, respiratory and cardiac motion represent distinct topological spaces. Therefore, as shown in Figure 19, we observed that the low‐frequency respiratory oscillations predominantly along the superior‐inferior and anterior‐posterior axes were modelled while the high‐frequency cardiac oscillations were largely ignored. Nonetheless, since the respiratory component dominates cardiac substructure motion, we found mean DSC >0.8 for the target and OARs with this approach. Future work will seek to extend *Voxelmap* to include this and other types of motion.

### 4.5 Online verification

Given that there is no ground‐truth for 3D internal organ motion during treatment, a practical challenge for *Voxelmap* involves online verification, *viz*. “How can we be sure that our model is performing well in real‐time?”. We offer two strategies for online verification. First, a pretreatment 4D scan (e.g. 4D‐CBCT) can be used to determine expected motion ranges for a given phase of the respiratory cycle. That is, the system could report whether *Voxelmap* exceeds the expected motion observed on the pretreatment scan. Second, the predicted contours could be forward‐projected onto the acquired kV projections (or MR images) so that the clinical team may verify the 2D positions of visible structures such as the lungs, heart, and diaphragm in real‐time.

## 5 CONCLUSIONS

A majority of radiation therapy centers around the world wish to offer real‐time respiratory motion monitoring to more patients but cannot afford to. *Voxelmap* addresses this problem by providing a software solution that can be inexpensively integrated into existing clinical workflows. Moreover, *Voxelmap* can be adapted to alternative image modalities as new treatment systems are developed. Here we validated this framework on digital phantom and patient data for cardiac radioablation and lung cancer treatments, demonstrating that target and organ‐at‐risk volumes can be predicted with Dice similarity >0.8 and 3D images can be predicted with structural similarity >0.9. However, certain network architectures were more robust to the scatter and noise profiles encountered in typical clinical settings, suggesting important directions for future developments.

## CONFLICT OF INTEREST STATEMENT

NH has filed a US patent for the method disclosed in this paper.

## Supporting information



Supporting Information

Supporting Information

Supporting Information

Supporting Information
