# Supplementary material for: An open‐source deep learning framework for respiratory motion monitoring and volumetric imaging during radiation therapy
Source: Med Phys. 2025 Jul 15;52(7):e18015. doi: 10.1002/mp.18015 (PMC12264095; doi:10.1002/mp.18015)
Supplement: Supplementary file 1 — Supporting Information [file MP-52-0-s004.docx]

For brevity, in the main text we included the results for the Dice similarity between ground-truth and predicted volumes averaged over all organs-at-risk for the XCAT. Below are the results for every structure in Table S-1.

Table s-1

|  | Network A | Network B | Network C |
| --- | --- | --- | --- |
| Patient 1 | | | |
| Target | 0.84 ± 0.08 | 0.80 ± 0.07 | 0.81 ± 0.07 |
| Stomach | 0.69 ± 0.07 | 0.86 ± 0.07 | 0.91 ± 0.06 |
| Esophagus | 0.68 ± 0.06 | 0.83 ± 0.06 | 0.86 ± 0.06 |
| Left Lung | 0.89 ± 0.02 | 0.95 ± 0.02 | 0.96 ± 0.02 |
| Right Lung | 0.89 ± 0.02 | 0.95 ± 0.02 | 0.96 ± 0.02 |
| Spinal cord | 0.79 ± 0.07 | 0.97 ± 0.00 | 0.88 ± 0.03 |
| Patient 2 | | | |
| Target | 0.85 ± 0.04 | 0.84 ± 0.04 | 0.85 ± 0.04 |
| Stomach | 0.87 ± 0.03 | 0.84 ± 0.02 | 0.88 ± 0.04 |
| Esophagus | 0.59 ± 0.09 | 0.46 ± 0.04 | 0.83 ± 0.04 |
| Left Lung | 0.91 ± 0.02 | 0.88 ± 0.02 | 0.94 ± 0.02 |
| Right Lung | 0.91 ± 0.02 | 0.89 ± 0.02 | 0.94 ± 0.02 |
| Spinal cord | 0.51 ± 0.11 | 0.26 ± 0.03 | 0.90 ± 0.00 |
| Patient 3 | | | |
| Target | 0.85 ± 0.02 | 0.85 ± 0.02 | 0.85 ± 0.02 |
| Stomach | 0.66 ± 0.07 | 0.65 ± 0.07 | 0.80 ± 0.08 |
| Esophagus | 0.70 ± 0.04 | 0.53 ± 0.07 | 0.89 ± 0.03 |
| Left Lung | 0.88 ± 0.02 | 0.87 ± 0.02 | 0.95 ± 0.02 |
| Right Lung | 0.88 ± 0.02 | 0.87 ± 0.02 | 0.94 ± 0.02 |
| Spinal cord | 0.44 ± 0.06 | 0.46 ± 0.13 | 0.97 ± 0.01 |
| Patient 4 | | | |
| Target | 0.86 ± 0.02 | 0.83 ± 0.04 | 0.86 ± 0.03 |
| Stomach | 0.84 ± 0.10 | 0.78 ± 0.11 | 0.73 ± 0.10 |
| Esophagus | 0.86 ± 0.02 | 0.58 ± 0.02 | 0.70 ± 0.03 |
| Left Lung | 0.95 ± 0.01 | 0.89 ± 0.01 | 0.90 ± 0.02 |
| Right Lung | 0.95 ± 0.01 | 0.90 ± 0.02 | 0.90 ± 0.02 |
| Spinal cord | 0.91 ± 0.03 | 0.25 ± 0.03 | 0.40 ± 0.07 |
| Patient 5 | | | |
| Target | 0.78 ± 0.06 | 0.77 ± 0.07 | 0.78 ± 0.06 |
| Stomach | 0.92 ± 0.03 | 0.94 ± 0.02 | 0.83 ± 0.04 |
| Esophagus | 0.84 ± 0.04 | 0.83 ± 0.06 | 0.55 ± 0.07 |
| Left Lung | 0.94 ± 0.02 | 0.94 ± 0.02 | 0.87 ± 0.02 |
| Right Lung | 0.95 ± 0.02 | 0.95 ± 0.01 | 0.87 ± 0.02 |
| Spinal cord | 0.96 ± 0.01 | 0.88 ± 0.02 | 0.35 ± 0.07 |
| Patient 6 | | | |
| Target | 0.80 ± 0.05 | 0.80 ± 0.05 | 0.80 ± 0.05 |
| Stomach | 0.71 ± 0.12 | 0.82 ± 0.11 | 0.68 ± 0.06 |
| Esophagus | 0.47 ± 0.06 | 0.66 ± 0.07 | 0.15 ± 0.09 |
| Left Lung | 0.85 ± 0.03 | 0.90 ± 0.03 | 0.81 ± 0.02 |
| Right Lung | 0.85 ± 0.03 | 0.90 ± 0.03 | 0.83 ± 0.02 |
| Spinal cord | 0.05 ± 0.01 | 0.45 ± 0.13 | 0.02 ± 0.04 |
| Patient 7 | | | |
| Target | 0.81 ± 0.04 | 0.81 ± 0.04 | 0.81 ± 0.04 |
| Stomach | 0.85 ± 0.05 | 0.81 ± 0.05 | 0.85 ± 0.05 |
| Esophagus | 0.85 ± 0.05 | 0.80 ± 0.05 | 0.82 ± 0.05 |
| Left Lung | 0.93 ± 0.02 | 0.91 ± 0.02 | 0.93 ± 0.02 |
| Right Lung | 0.93 ± 0.02 | 0.91 ± 0.02 | 0.93 ± 0.02 |
| Spinal cord | 0.96 ± 0.00 | 0.86 ± 0.04 | 0.89 ± 0.01 |
| Patient 8 | | | |
| Target | 0.79 ± 0.05 | 0.78 ± 0.06 | 0.77 ± 0.06 |
| Stomach | 0.68 ± 0.12 | 0.63 ± 0.09 | 0.88 ± 0.08 |
| Esophagus | 0.57 ± 0.17 | 0.63 ± 0.07 | 0.88 ± 0.05 |
| Left Lung | 0.89 ± 0.02 | 0.90 ± 0.02 | 0.96 ± 0.02 |
| Right Lung | 0.89 ± 0.02 | 0.89 ± 0.02 | 0.96 ± 0.02 |
| Spinal cord | 0.52 ± 0.29 | 0.57 ± 0.07 | 0.88 ± 0.02 |
