# Supplementary material for: An open‐source deep learning framework for respiratory motion monitoring and volumetric imaging during radiation therapy
Source: Med Phys. 2025 Jul 15;52(7):e18015. doi: 10.1002/mp.18015 (PMC12264095; doi:10.1002/mp.18015)
Supplement: Supplementary file 2 — Supporting Information [file MP-52-0-s002.docx]

For brevity, in the main text we included the results for the Dice similarity between ground-truth and predicted volumes averaged over all organs-at-risk for the CoMBAT. Below are the results for every structure in Table S-2.

Table s-2

|  | Network E | Network F |
| --- | --- | --- |
| Patient 1 | | |
| Target | 0.83 ± 0.08 | 0.82 ± 0.08 |
| Stomach | 0.82 ± 0.02 | 0.82 ± 0.01 |
| Esophagus | 0.82 ± 0.01 | 0.82 ± 0.01 |
| Left Lung | 0.94 ± 0.01 | 0.94 ± 0.01 |
| Right Lung | 0.94 ± 0.01 | 0.94 ± 0.01 |
| Spinal cord | 0.95 ± 0.01 | 0.96 ± 0.00 |
| Patient 2 | | |
| Target | 0.85 ± 0.04 | 0.84 ± 0.04 |
| Stomach | 0.88 ± 0.01 | 0.88 ± 0.02 |
| Esophagus | 0.84 ± 0.01 | 0.83 ± 0.02 |
| Left Lung | 0.93 ± 0.01 | 0.93 ± 0.02 |
| Right Lung | 0.93 ± 0.01 | 0.93 ± 0.01 |
| Spinal cord | 0.98 ± 0.01 | 0.97 ± 0.01 |
| Patient 3 | | |
| Target | 0.83 ± 0.04 | 0.83 ± 0.04 |
| Stomach | 0.81 ± 0.05 | 0.84 ± 0.05 |
| Esophagus | 0.89 ± 0.02 | 0.90 ± 0.02 |
| Left Lung | 0.95 ± 0.01 | 0.95 ± 0.02 |
| Right Lung | 0.94 ± 0.01 | 0.95 ± 0.02 |
| Spinal cord | 0.96 ± 0.01 | 0.97 ± 0.01 |
| Patient 4 | | |
| Target | 0.85 ± 0.03 | 0.85 ± 0.03 |
| Stomach | 0.93 ± 0.02 | 0.92 ± 0.02 |
| Esophagus | 0.90 ± 0.04 | 0.90 ± 0.03 |
| Left Lung | 0.97 ± 0.01 | 0.97 ± 0.01 |
| Right Lung | 0.97 ± 0.01 | 0.97 ± 0.01 |
| Spinal cord | 0.93 ± 0.06 | 0.93 ± 0.06 |
| Patient 5 | | |
| Target | 0.77 ± 0.08 | 0.76 ± 0.09 |
| Stomach | 0.95 ± 0.01 | 0.95 ± 0.01 |
| Esophagus | 0.87 ± 0.05 | 0.87 ± 0.05 |
| Left Lung | 0.95 ± 0.01 | 0.96 ± 0.01 |
| Right Lung | 0.96 ± 0.01 | 0.96 ± 0.01 |
| Spinal cord | 0.96 ± 0.01 | 0.97 ± 0.01 |
| Patient 6 | | |
| Target | 0.75 ± 0.07 | 0.78 ± 0.07 |
| Stomach | 0.91 ± 0.03 | 0.90 ± 0.04 |
| Esophagus | 0.80 ± 0.04 | 0.85 ± 0.03 |
| Left Lung | 0.93 ± 0.02 | 0.94 ± 0.01 |
| Right Lung | 0.94 ± 0.02 | 0.94 ± 0.01 |
| Spinal cord | 0.87 ± 0.08 | 0.92 ± 0.00 |
| Patient 7 | | |
| Target | 0.74 ± 0.07 | 0.76 ± 0.07 |
| Stomach | 0.88 ± 0.03 | 0.86 ± 0.03 |
| Esophagus | 0.81 ± 0.03 | 0.85 ± 0.04 |
| Left Lung | 0.94 ± 0.01 | 0.94 ± 0.02 |
| Right Lung | 0.94 ± 0.01 | 0.94 ± 0.02 |
| Spinal cord | 0.92 ± 0.01 | 0.96 ± 0.00 |
| Patient 8 | | |
| Target | 0.77 ± 0.06 | 0.76 ± 0.07 |
| Stomach | 0.79 ± 0.04 | 0.79 ± 0.06 |
| Esophagus | 0.85 ± 0.03 | 0.83 ± 0.04 |
| Left Lung | 0.95 ± 0.01 | 0.94 ± 0.02 |
| Right Lung | 0.95 ± 0.01 | 0.94 ± 0.02 |
| Spinal cord | 0.96 ± 0.02 | 0.92 ± 0.00 |
