# Supplementary material for: An open‐source deep learning framework for respiratory motion monitoring and volumetric imaging during radiation therapy
Source: Med Phys. 2025 Jul 15;52(7):e18015. doi: 10.1002/mp.18015 (PMC12264095; doi:10.1002/mp.18015)
Supplement: Supplementary file 3 — Supporting Information [file MP-52-0-s001.docx]

To assess the robustness of learned representations to noise, we performed a feature correlation analysis of encoder activations across increasing levels of Poisson noise for Networks A, B, and C. This analysis quantifies how similarly each model encodes input data under noise perturbations and provides insight into the stability of internal representations.

**Data Preparation:**
We used the XCAT data with the same configuration described in the main manuscript. Poisson noise was synthetically added to the target 2D projection image using a λ parameter corresponding to expected photon counts (λ = 50, 100, 200), simulating progressively lower-dose acquisition conditions. A “no noise” condition (λ = 0) served as the reference.

**Feature Extraction:**
For each network, we registered a forward hook on all layers of the 2D encoding arm (i.e., layers responsible for processing x-ray projections). We passed all test samples through the network under each noise condition and collected the output feature activations from the encoder. These were flattened and concatenated across the test set to form a large matrix of activations for each noise level.

**Correlation Computation:**
For each model, we computed the Pearson correlation coefficient between encoder activations obtained under the no-noise condition and those obtained under each Poisson noise level (50, 100, 200). This resulted in a correlation value that captures the similarity between clean and noisy internal representations. A higher value indicates greater representational stability under noise.

Table s-3

| **Network** | **Poisson λ= 50** | **Poisson λ=100** | **Poisson λ=200** |  |
| --- | --- | --- | --- | --- |
| **A** | 0.842 | 0.880 | 0.943 |  |
| **B** | 0.364 | 0.496 | 0.818 |  |
| **C** | 0.350 | 0.564 | 0.716 |  |

Table S-3 summarizes the correlation values for each model and noise level. These results highlight that Network A maintains a high degree of activation similarity even under increasing noise, suggesting that early fusion of 2D projections leads to more robust and consistent feature encoding. In contrast, Networks B and C exhibit more noise-sensitive representations, particularly at lower noise levels. Increasing correlations with more noise, may might seem counterintuitive. However, at high noise levels, finer spatial detail is lost, so the encoder resorts to more generic features, which can become similar across all inputs, leading to higher correlation.
