# Supplementary material for: An open‐source deep learning framework for respiratory motion monitoring and volumetric imaging during radiation therapy
Source: Med Phys. 2025 Jul 15;52(7):e18015. doi: 10.1002/mp.18015 (PMC12264095; doi:10.1002/mp.18015)
Supplement: Supplementary file 4 — Supporting Information [file MP-52-0-s003.docx]

One difference in training *Voxelmap* on x-ray vs MRI data is that training examples can be abundantly generated in the former case by forward-projecting the 4D-CT in multiple projection angles. To create a similarly abundant training dataset for the MRI guided treatments, we used off-center slices. As shown in Figure S1, we observed that using the central 16 × 16 slice pairs (yielding 16 × 16 × 10 = 2560 training examples), network performance converged efficiently with similar performance to using the central 32 × 32 slice pairs and much faster training times (5h vs 21h).


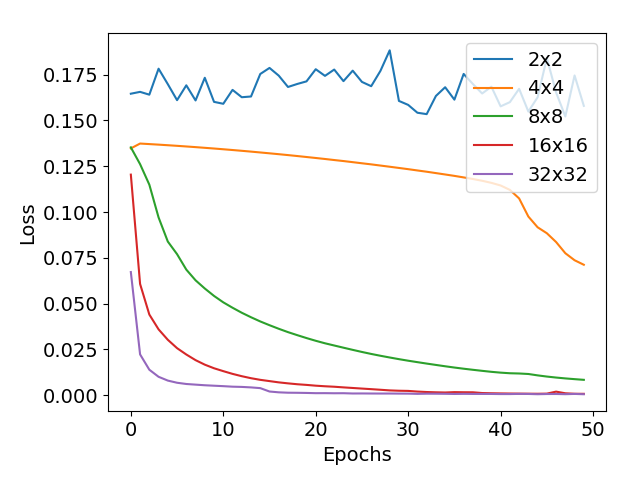


Fig. S1. Loss curves for Network D using the central 2×2, 4×4, 8×8, 16×16, or 32×32 coronal-sagittal slice pairs.

For brevity, in the main text we included the results for the Dice similarity between ground-truth and predicted volumes averaged over all organs-at-risk for the XCAT and CoMBAT data. Below are the results for every structure in Tables S1 and S2.
